# ZFPM2-AS1 promotes the proliferation, migration, and invasion of human non-small cell lung cancer cells involving the JAK-STAT and AKT pathways

**DOI:** 10.7717/peerj.10225

**Published:** 2020-10-26

**Authors:** Xiwen Wang, Jun Tang, Jungang Zhao, Bin Lou, Li Li

**Affiliations:** 1 Department of Thoracic Surgery, Shengjing Hospital of China Medical University, Shenyang, Liaoning, China; 2 Department of Hygiene Toxicology, School of Public Health, China Medical University, Shenyang, Liaoning, China; 3 Department of Gerontology and Geriatrics, Shengjing Hospital of China Medical University, Shenyang, Liaoning, China

**Keywords:** NSCLC, LncRNA, ZFPM2-AS1, Immune infiltration, PD-L1

## Abstract

**Purpose:**

Recent studies have determined that long non-coding RNAs (lncRNAs) are potential prognostic biomarkers for non-small cell lung cancers (NSCLCs). The purpose of this study was to analyze the function and associated pathways of zinc finger protein multitype 2 antisense RNA 1 (ZFPM2-AS1) in NSCLC cells.

**Methods:**

We used qRT-PCR to analyze ZFPM2-AS1’s transcription level. Its proliferation, migration, and invasion capacities were determined using MTT, colony forming, wound healing, and transwell assays. We additionally analyzed the correlation between ZFPM2 and immune infiltration using the Tumor Immune Estimation Resource (TIMER) database, and the protein expression levels using Western blots.

**Results:**

We found that ZFPM2-AS1 expression in NSCLC specimens and cell lines was elevated compared to the control group. ZFPM2-AS1 is an oncogene and independent prognostic predictor of poor survival in NSCLCs, and its expression had a positive correlation with tumor size and lymph node metastasis in our clinical data. MTT, colony forming, wound healing, and transwell assays showed a positive correlation between ZFPM2-AS1 expression and the proliferation, migration, and invasion of NSCLC cells in the presence and absence of interferon- (IFN-*γ*). Using the TIMER database, we hypothesized that ZFPM2 was negatively correlated with ZFPM2-AS1 expression, as well as the immune infiltration levels in lung adenocarcinoma (LUAD). Finally, we found that ZFPM2-AS1 negatively regulated ZFPM2 expression, and had a positive correlation with PD-L1 expression through the JAK-STAT and AKT pathways.

**Conclusion:**

Our study confirmed that ZFPM2-AS1 promotes the proliferation, migration, and invasion of NSCLC cells via the JAK-STAT and AKT pathways. Further research on the ZFPM2-AS1 pathway regulation mechanism is needed.

## Introduction

Lung cancer is the most deadly malignant tumor, accounting for more than 80% of non-small cell lung cancers (NSCLCs) worldwide, most of which are lung adenocarcinoma (LUAD) and lung squamous cell carcinoma (LUSC). Multiple therapies have improved the prognosis of NSCLCs, but the 5-year survival rate has remained lower than 20% (Hirsch et al., 2017). Therefore, it is crucial to explore novel diagnostic biomarkers and therapeutic approaches (Ma et al., 2012; Han et al., 2015).

Long non-coding RNAs (lncRNAs), defined as non-coding transcripts longer than 200 nucleotides, are associated with the initiation, progression, and prognosis of various tumors (Du et al., 2013; Prensner & Chinnaiyan, 2011). Mounting dysregulated lncRNAs may also play a role as tumor suppressors or oncogenes in multiple tumors, including NSCLCs.

We previously screened differential expression lncRNAs in NSCLCs using The Cancer Genome Atlas (TCGA) database (Wang et al., 2019b). We speculated that one of the most distinct lncRNAs, zinc finger protein multitype 2 antisense RNA 1 (ZFPM2-AS1), may play an important role in the biological behavior of NSCLC cells. In this study, we concentrated on the functions of ZFPM2-AS1. ZFPM2-AS1 reportedly induces p53 destabilization by stabilizing macrophage migration inhibitory factor (MIF), leading to the progression of gastric cancer (Kong et al., 2018). ZFPM2-AS1 has also been shown to promote metastasis and proliferation, as well as inhibit renal cell cancer apoptosis by targeting miR-137 (Liu et al., 2019). Additionally, ZFPM2-AS1 can promote NSCLC progression via the miR-511-3p/AFF4 and miR-18b-5p/VMA21 pathways (Li et al., 2020; Xue et al., 2020), and enhance the malignancy of cervical cancer by sponging microRNA-511-3p (Dai et al., 2020). By upregulating TRAF4, ZFPM2-AS1 facilitates cell proliferation in both esophageal squamous cell carcinoma and small cell lung cancer (Sun & Wu, 2020; Yan et al., 2020). However, ZFPM2-AS1’s molecular regulatory network in NSCLC cells remains unclear.

The PI3K/AKT and JAK/STAT signaling pathways promote cell proliferation and motility by activating key metastasis-promoting genes (Teng, Ross & Cowell, 2014). STAT activation is restricted in normal cells. However, once STAT is activated, numerous genes that control tumor cell proliferation, angiogenesis, and evasion of immune surveillance are uncontrollably expressed (Bowman et al., 2000). Interferon- *γ* (IFN-*γ*) is crucial for immunity against intracellular pathogens and tumor cells (Schoenborn & Wilson, 2007). Since IFN-*γ* has the ability to induce PD-L1, IFN-*γ* expression in cancer cells may weaken the immunity of specific tumor cells (Abiko et al., 2015). Additionally, it has been found that PD-L1 expression is positively correlated with JAK2 in NSCLCs via the JAK-STAT axis (Ikeda et al., 2016). However, it has not been proved whether ZFPM2-AS1 can regulate PD-L1 via the JAK-STAT and AKT pathways.

In this study, we investigated ZFPM2-AS1’s proliferation, migration, and invasion abilities in NSCLC cells. We also determined the regulatory roles of ZFPM2-AS1 in the JAK-STAT and AKT pathways.

## Materials and Methods

### TCGA and Tumor Immune Estimation Resource (TIMER) databases

We used ZFPM2-AS1 transcript expression levels extracted from TCGA’s database for our fragments per kilobase million (FPKM) values. The FPKM values were plotted in a scatterplot and on receiver operating characteristic (ROC) curves. We used OmicShare tools (http://www.omicshare.com/tools) to find the area under the ROC curve (AUC) in order to estimate the diagnostic values (sensitivity and specificity). TIMER (https://cistrome.shinyapps.io/timer) is a novel database that includes 10,897 samples across 39 tumor types from TCGA (Li et al., 2017), along with specific genes’ tumor immune infiltration levels. We analyzed ZFPM2 expression across multiple tumor types using the different expression module, and identified the association between ZFPM2 expression and immune infiltration level using the gene module.

### Patients and samples

Surgical specimens were collected from 50 individual patients undergoing NSCLC surgery at the Affiliated Shengjing Hospital of China Medical University (Shenyang, China) between May 2017 and August 2018. All specimens had been pathologically diagnosed as LUAD or LUSC. The specimens were frozen at −80 °C directly following surgery. Our experimental protocol was authorized by the Shengjing Hospital Ethics Committee (2018PS170K), and we acquired written informed consent from each patient.

### Cell culture, reagent, and transfection

The human NSCLC cell lines (A549 and H460) were purchased from the Shanghai Institutes of Biochemistry and Cell Biology, Chinese Academy of Sciences, Shanghai, China. The A549 and H460 cell lines had been cultured in RPMI-1640 with 10% fetal bovine serum (Clark Biosciences, Richmond, VA, USA), 100 U/ml penicillin, and 100 ug/ml streptomycin (Sigma-Aldrich, St. Louis, MO, USA) in a 5% CO_2_ incubator at 37 °C. During IFN- *γ* stimulation, cells were incubated with 100 ng/ml of recombinant human IFN-*γ* (Peprotech, Cranbury, NJ, USA) for 48 h. We used Lipo3000 (Invitrogen, Carlsbad, CA, USA) according to our transfection protocol. We used 20 uM of lncRNA Smart Silencer (RiboBio, Guangzhou, China) and a mixture of three siRNAs and three antisense oligonucleotides. The sequences are provided in Table S1. ZFPM2-AS1 overexpression plasmid was provided by GenePharma (Shanghai, China).

### RNA isolation, cDNA synthesis, and quantitative real-time RT-PCR

We extracted total RNA using TRIzol reagent, and performed reverse-transcription using HisScript™ QRT SuperMix (Vazyme Biotech Co., Ltd., Nanjing, China). We used qRT-PCR and ChamQ™ Universal SYBR qPCR Master Mix (Vazyme Biotech Co., Ltd.) to analyze the relative expression of the control group. The specific primers are shown in Table S1. GAPDH and RPS18 were used as housekeeping genes. The relative gene expression was calculated using the 2^−ΔΔ*Cq*^ method.

### MTT assay

Cell viability was determined using MTT reagent (Sigma-Aldrich) with a concentration of 0.5 mg/mL for 4 h. We seeded 4 ×10^3^ cells per well in 96-well plates for 0, 1, 2, 3, 4, and 5 days. The supernatant was abandoned, and we precipitated formazan with DMSO. Finally, we analyzed the absorbance at 450 nm using a microplate reader (Thermo Fisher Scientific, Waltham, MA, USA).

### Colony formation assay

We cultured about 3,000 terminated trypsinized cells in 6-cm dishes three times at 37  °C in 5% CO_2_. Two weeks later, the cell colonies were fixed with 10% methanol for 30 s, and then stained with 0.1% crystal violet (Sigma-Aldrich) for 15 min. Finally, the visible colonies were counted using a microscope.

### Wound healing assay

A549 and H460 cells were seeded into 5 ×10^5^cells/well in six-well plates three times. A 200 *μ*L pipette tip was used to make a scratch where the confluence reached 90%. The cells were then incubated at 37 ° C in 5% CO_2_ for 24 h. We studied the migration distances using an FSX100 Biological Image system (Olympus, Tokyo, Japan).

### Transwell migration and invasion assay

Migration and invasion assays were placed in 24-well Transwell chambers that had 8 µm size pores (Costar, Washington, D.C., USA). In the invasion assay, the pores were covered with 100 µL of Matrigel (BD Biosciences, San Jose, CA, USA). After trypsinization, we placed 100 µL of medium (5 ×10^4^ cells) supplemented with 2% fetal bovine serum in the upper Transwell chamber, and 600 µL of medium supplemented with 10% fetal bovine serum in the lower chamber. After 24 h of incubation, the upper cells were removed, and the lower ones were fixed with paraformaldehyde and stained with hematoxylin. The number of migrated/invaded cells was analyzed using 10 randomly selected fields at ×200 magnification under phase contrast microscopy (Olympus). All assays were performed independently at least three times.

### Nuclear-cytoplasmic localization

We harvested and washed the A549 cells before adding 500 µl of cell disruption buffer (PARIS kit; cat. no. AM1921; Invitrogen/Thermo Fisher Scientific) to the cells on ice for 10 min. Following centrifugation at 500 × g, the supernatants were preserved as cytoplasmic RNA. They were washed, an equal volume of nuclear lysate buffer was added, and they were centrifuged at 500×g. Finally, the supernatants were collected as nuclear RNA and we performed qRT-PCR using the primers listed in Table S1.

### Flow cytometry

For cell cycle analysis, we harvested the transfected cells, washed them with PBS, resuspended them in 0.9 ml of PBS, and gradually added ice-cold ethanol up to a volume of 3 ml. After 24 h of fixation, the cells were incubated with 0.1% Triton X-100, 0.2 mg/ml RNase A, and 25 µg/ml propidium iodide (PI) for 30 min at room temperature. We used flow cytometry to assay the DNA content (Becton Dickinson, Bedford, MA, USA), and ModFit software (Verify Software) to quantify the percentage of cells within the S-, G0/G1-, and G2/M- phases of the cell cycle.

### Western blot analysis

After harvesting the proteins using a lysis buffer (50 mM of Tris (pH 7.4), 1% Triton X-100, 0.5% Nonidet P-40, 150 mM NaCl, and protease inhibitor), we separated them using 10% sodium dodecyl sulfate (SDS) polyacrylamide gel electrophoresis. The proteins were then transferred to polyvinylidene difluoride (PVDF) membranes (Millipore, Burlington, MA, USA) for 2 h at 220 mA. The membranes were blocked in 5% BSA with TBST for 1 h at room temperature, and were incubated overnight using primary antibodies at 4  °C. We incubated the secondary antibody with horseradish peroxidase (HRP) conjugates for 1 h at room temperature. We identified the bands using a chemiluminescence detection kit (Tanon, Shanghai, China). We found that the primary antibodies included PDL1 (E1L3N), JAK2 (D2E12), phospho-STAT3 (Tyr705; D3A7), STAT3 (79D7), phospho-AKT (Ser473; D9E), AKT (40D4; all purchased from Cell Signaling Technology, Boston, MA, USA), and ZFPM2 (OriGene).

### Statistical analysis

The log-rank test was performed using the Kaplan–Meier survival analysis procedure (Schoenborn & Wilson, 2007). We conducted statistical analyses with GraphPad Prism7 (GraphPad Software, Inc., San Diego, CA, USA). Test data were manifested as means ± standard deviation (SD). We used the student’s *t*-test (two-tailed) to find the differences between two groups, and one-way ANOVA to find the differences across more than two groups, followed by Dunnett’s post-test. *P* < 0.05 was considered statistically significant.

## Results

### Increased ZFPM2-AS1 related to poor survival in in NSCLC patients

**Figure 1:**
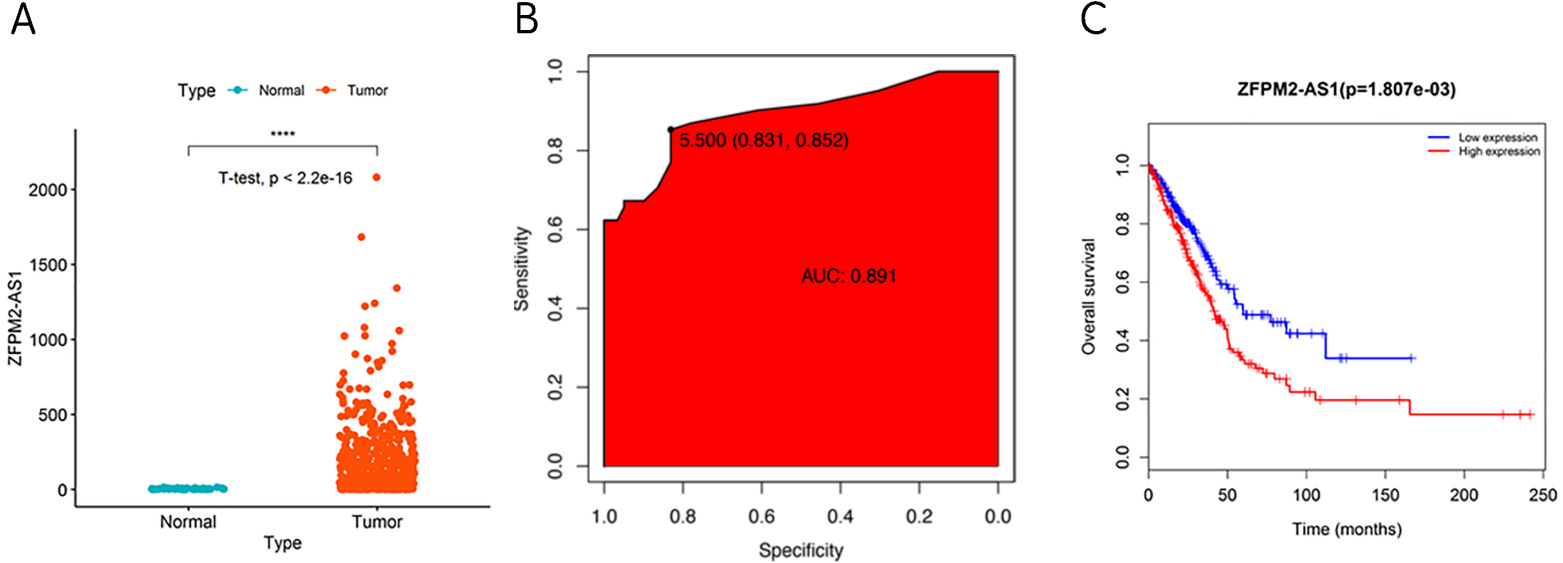
Correlation between higher ZFPM2-AS1 expression and poor prognosis. (A) Scatter plot of ZFPM2-AS1 expression values in NSCLC and normal tissue samples from the RNA-Seq dataset of TCGA (y-axis represents the FPKM value, **** means p<0.0001). (B) AUC value of the ROC curve based on ZFPM2-AS1 expression values. (C) The Kaplan–Meier survival curve with its corresponding log-rank test of ZFPM2-AS1.

During our investigation, we used ZFPM2-AS1 RNA-seq data from TCGA to analyze the differential expressions across 59 normal lung tissues and 535 LUADs or LUSCs. We found a significant increase of ZFPM2-AS1 in NSCLCs compared to normal lung tissues (Fig. 1A). Furthermore, when analyzing the ROC curve based on the screened TCGA data, we found that ZFPM2-AS1′s AUC value was 0.891 (Fig. 1B), indicating that ZFPM2-AS1 may be a novel diagnostic biomarker. Generated using screened data from TCGA, the Kaplan–Meier survival curve showed that higher ZFPM2-AS1 expression levels were significantly associated with poor prognoses for NSCLC patients (Fig. 1C). Ultimately, our results suggested that ZFPM2-AS1 is a possible oncogene in NSCLCs.

### Validating ZFPM2-AS1 expression patterns in NSCLC samples and cell lines

**Figure 2:**
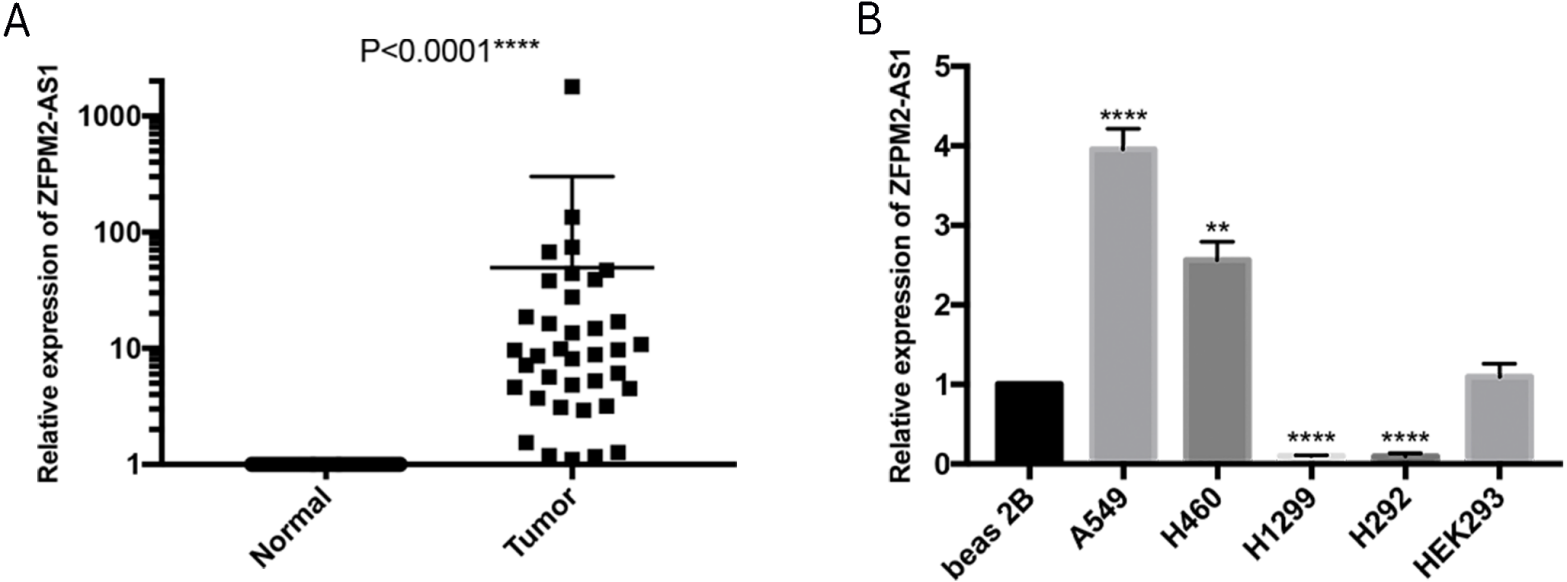
ZFPM2-AS1 upregulation in NSCLC samples and cell lines. The expression levels of samples and cell lines were determined using quantitative RT-PCR. All data analyses were performed using the mean values of individual tissues or the mean value ±SD of each cell line from three independent experiments. (A) Differential ZFPM2-AS1 expression across 50 NSCLC pairs and adjacent normal tissues. An unpaired Student’s t-test was used to find this statistical result. (B) Differential ZFPM2-AS1 expression in individual cell lines. The statistical result was found using ANOVA (parametric) test. * P<0.05, ** P<0.01, *** P<0.001, **** P<0.0001.

To further verify the ZFPM2-AS1 expression patterns that we found in TCGA’s RNA-Seq databases, we used qRT-PCR to validate 50 pairs of collected NSCLC and adjacent normal samples. We found that the relative transcription expression levels were significantly higher in NSCLC tissue compared to the adjacent normal tissues (Fig. 2A; *P* < 0.0001). We also validated BEAS-2B, A549, NCI-H460, H1299, H292, and HEK293 cells by performing qRT-PCR, and found that ZFPM2-AS1 was expressed in all of these cell lines. Compared to the BEAS-2B cell expression levels, ZFPM2-AS1 was significantly upregulated in the A549 and H460 cells. The expression levels were significantly downregulated in the NSCLC cell lines H1299 and H292 (Fig. 2B). These results implied that ZFPM2-AS1′s function in A549 and H460 cells may be similar to its functions in NSCLC samples.

### The association between ZFPM2-AS1 and NSCLC clinical characteristics

When examining the possible association between ZFPM2-AS1 expression levels and the clinical parameters of 50 NSCLC patients, we found that tumor size was significantly positively correlated with ZFPM2-AS1 expression (*p* = 0.007). The tumor-node-metastasis (TNM) stage was also positively correlated with ZFPM2-AS1 expression (*p* = 0.047). However, ZFPM2-AS1 expression did not have a significant correlation with other clinical characteristics, including age, gender, tumor differentiation, smoking history, and lymph node metastasis (Table 1). Our results showed that ZFPM2-AS1 expression was positively correlated to tumor size and TNM stage.

**Table 1:** The association between ZFPM2-AS1 expression and clinical measures in LUAD patients.

Characteristics	N	Relative ZFPM2-AS1 expression		
		Low	High	*P* value
Age(years)				
>65	11	5	6	0.733
≤65	39	20	19	
Gender				
Male	24	14	10	0.258
Female	26	11	15	
Differentiation				
Well,moderate	38	19	19	0.999
Poor	12	6	6	
Tumor size (maximum diameter)				
>3 cm	33	12	21	0.007^**^
≤3 cm	17	13	4	
Histological tumor type				
Squamous cell carcinoma	20	8	12	0.248
Adenocarcinoma	30	17	13	
Smoking history				
Smokers	20	11	9	0.564
Never smokers	30	14	16	
Lymph node metastasis				
Positive	23	11	12	0.777
Negative	27	14	13	
TNM stage				
1stage	23	15	8	0.047^*^
2.3.4stage	27	10	17	

**Notes.**

Data were analyzed by Chi-square. Median value: 5.04.

* *P* < 0.05.

** *P* < 0.01.

Data were analyzed by Chisquare test. The high and low groups were divided by the median expression value of ZFPM2AS1 (5.04).

### ZFPM2-AS1 knockdown decreases proliferation and colony formation in NSCLC cell lines

**Figure 3:**
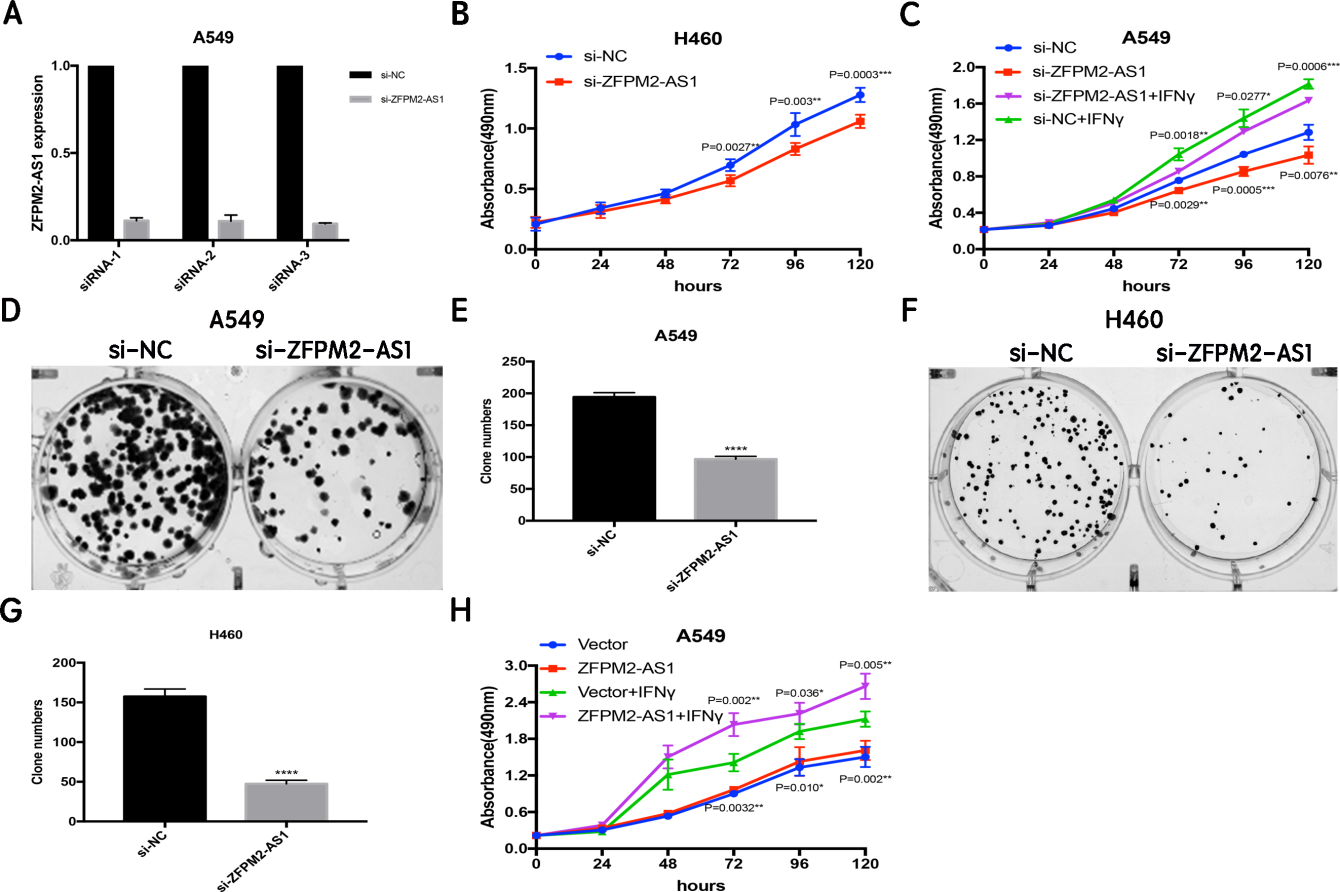
The positive correlation between ZFPM2-AS1 and NSCLC cell proliferation. (A) qRT-PCR was used to evaluate the knockdown efficiency (24h) of ZFPM2-AS1 with the specific siRNAs and scrambled target sequences. The y-axis value represented the ratio of ZFPM2-AS1 expression in siRNA groups to that of the control group. GAPDH was used as the reference gene. (B) The MTT assay was performed so that the ZFPM2-AS1 knockdown inhibited H460 cell proliferation. The statistical data were analyzed using an unpaired Student’s t-test. (C) ZFPM2-AS1 knockdown inhibited A549 cell proliferation with and without IFN-*γ*. (D-G) Colony formation assays were performed to illuminate A549 and H460 cell proliferation upon ZFPM2-AS1 knockdown. (H) The effect of ZFPM2-AS1 overexpression on A549 cell proliferation with and without IFN-*γ*. *P <0.05. **P <0.01. ***P <0.001. ****P <0.0001.

Since ZFPM2-AS1 was highly expressed in A549 cells, we used siRNA-mediated ZFPM2-AS1 knockdown in A549 cells to analyze ZFPM2-AS1′s biological functions. To minimize the off-target effects on lncRNA, we used specific Smart Silencers, including three individual siRNAs and three individual antisense oligonucleotides. The decreased efficiency was confirmed by qRT-PCR (Fig. 3A). Subsequently, we used an MTT assay to show any cell proliferation caused by ZFPM2-AS1 silencing or overexpression. We also stimulated ZFPM2-AS1 using IFN-*γ* because of its ability to induce PD-L1 and promote the immune escape of tumor cells. The results showed that the proliferation rate of ZFPM2-AS1 silencing in the H460 cells was aberrantly lower than in the control group after 72–120 h (Fig. 3B). In the A549 cells, the proliferation rate was significantly lower when silencing ZFPM2-AS1 after 72 h without IFN-*γ*, and after 96–120 h with IFN-*γ* (Fig. 3C). We obtained similar results when performing colony forming assays, confirming that ZFPM2-AS1 knockdown distinctly inhibited colony forming in both A549 and H460 cells (Figs. 3D–3G). Meanwhile, ZFPM2-AS1 overexpression significantly promoted A549 cell proliferation after 96–120 h without IFN-*γ*, and after 72–120 h with IFN-*γ* (Fig. 3H). These results suggest that ZFPM2-AS1′s role as an oncogene involves promoting NSCLC cell line proliferation.

### ZFPM2-AS1 promotes the migration and invasion of NSCLC cell lines

**Figure 4:**
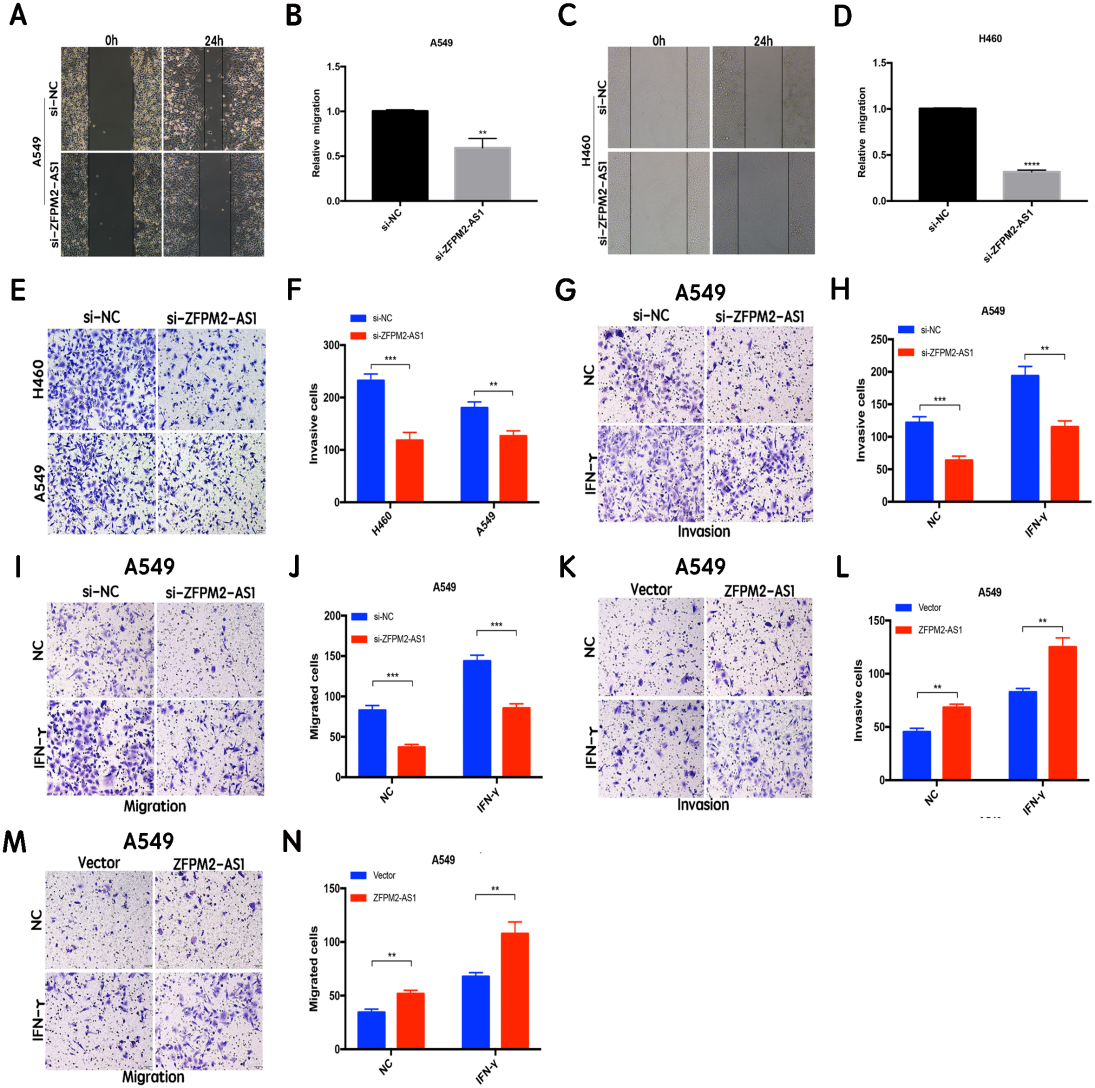
The positive correlation between ZFPM2-AS1 and the migration and invasion of NSCLC cells. (A-D) ZFPM2-AS1 knockdown inhibited the motility of A549 and H460 cells by the wound healing assay. (E) (F) The transwell assay was performed to determine whether ZFPM2-AS1 knockdown inhibited A549 and H460 cell invasion. (G-J) In A549 cells, ZFPM2-AS1 silencing decreased the invasion and migration potential with and without IFN-*γ* (48h). (K-N) The impact of ZFPM2-AS1 overexpression on the invasion and migration ability of A549 cells with and without IFN-*γ* (48h). *P <0.05. **P <0.01. ***P <0.001.

The results of the wound healing assay indicated that ZFPM2-AS1 knockdown significantly inhibited A549 and H460 cell motility when compared to the control group (Figs. 4A–4D). We used the Transwell assay to investigate whether migration and invasion were also affected by ZFPM2-AS1 in NSCLC cells. We found that the siRNA-mediated ZFPM2-AS1 knockdown significantly inhibited the invasion capacities of both A549 and H460 cells (Figs. 4E, 4F). The silencing of ZFPM2-AS1 distinctly impeded the invasion and migration capabilities of A549 cells, in both the presence and absence of IFN-*γ* (Figs. 4G–4J). Meanwhile, ZFPM2-AS1 overexpression significantly promoted invasion and migration in the presence or absence of IFN-*γ* (Figs. 4K–4N). Ultimately, we determined that ZFPM2-AS1 promoted migration and invasion in both A549 and H460 cells.

### ZFPM2-AS1′s primary expression in the nucleus did not affect the A549 cell cycle

**Figure 5:**
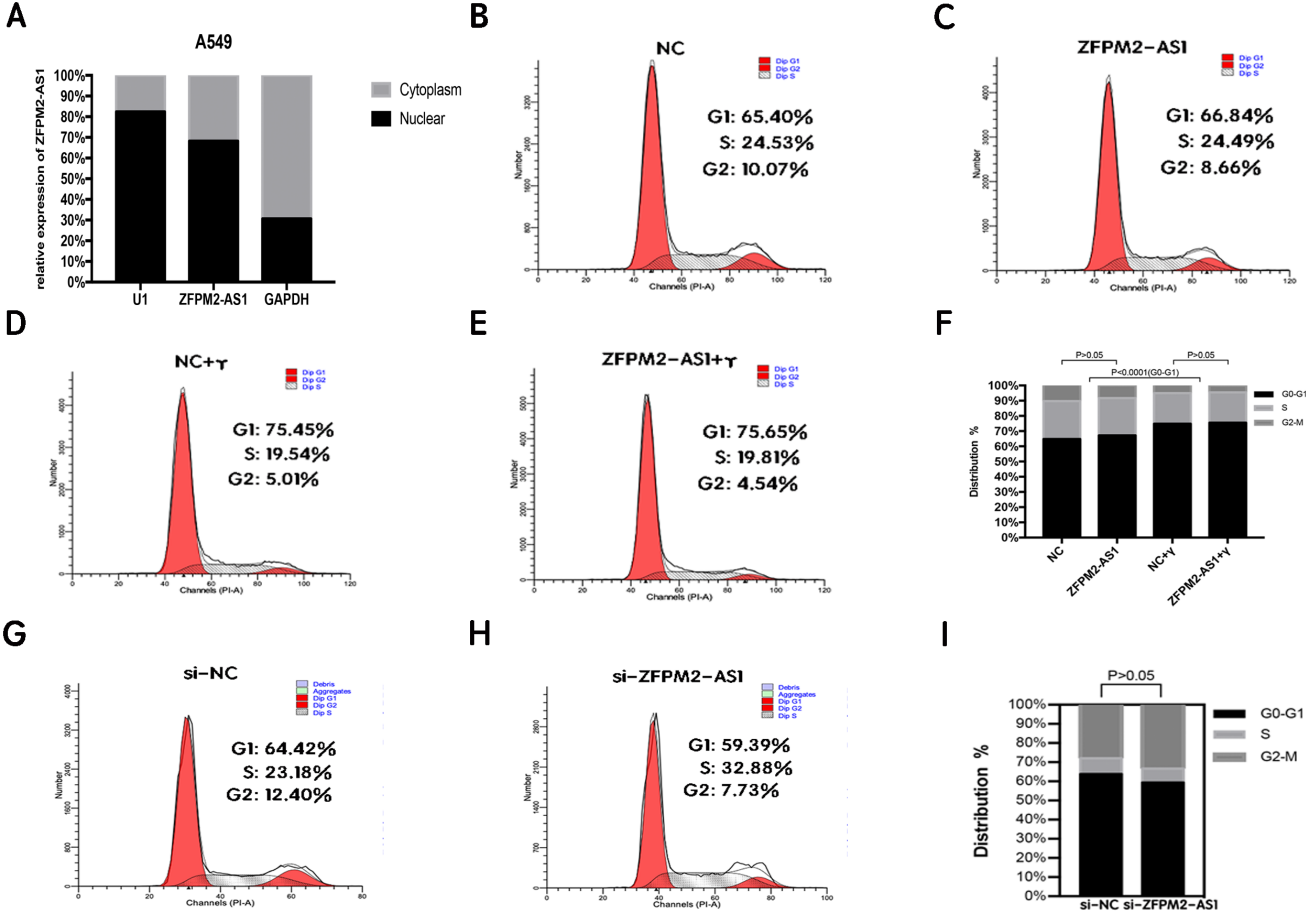
The location and effect on the ZFPM2-AS1 cell cycle. (A) The ZFPM2-AS1 expression levels in the A549 cell nucleus and cytoplasm fractions were found using qRT-PCR. The statistical Chi-square test was performed with three independent experiments. (B-I) We analyzed the flow cytometric of the cell cycle 24 hrs after ZFPM2-AS1 knockdown or overexpression in the presence or absence of IFN-*γ*.

To clarify its cellular localization, we used qRT-PCR to determine ZFPM2-AS1 expression in both the nuclear and cytoplasmic fractions of A549 cells. We used GAPDH expression for the cytoplasmic indicator, and U1 snRNA enrichment for the nuclear indicator. The results showed that ZFPM2-AS1 was primarily expressed in the nucleus (Fig. 5A), indicating that ZFPM2-AS1 may also regulate the functions of NSCLC cells in the nucleus. We used a flow cytometric analysis to determine whether ZFPM2-AS1 silencing or overexpression had an impact on the cell cycle, with and without IFN-*γ*. The results showed that IFN-*γ* arrested A549 cells at the G0/G1 phase. However, ZFPM2-AS1 silencing and overexpression did not significantly affect the A549 cell cycle (Figs. 5B–5I). Our results confirmed that ZFPM2-AS1 was primarily expressed in the nucleus, but its effect on the proliferation, migration, and invasion of A549 cells was not dependent on the cell cycle.

### The association between ZFPM2, a potential target for ZFPM2-AS1, and LUAD tumor immune infiltration level

**Figure 6:**
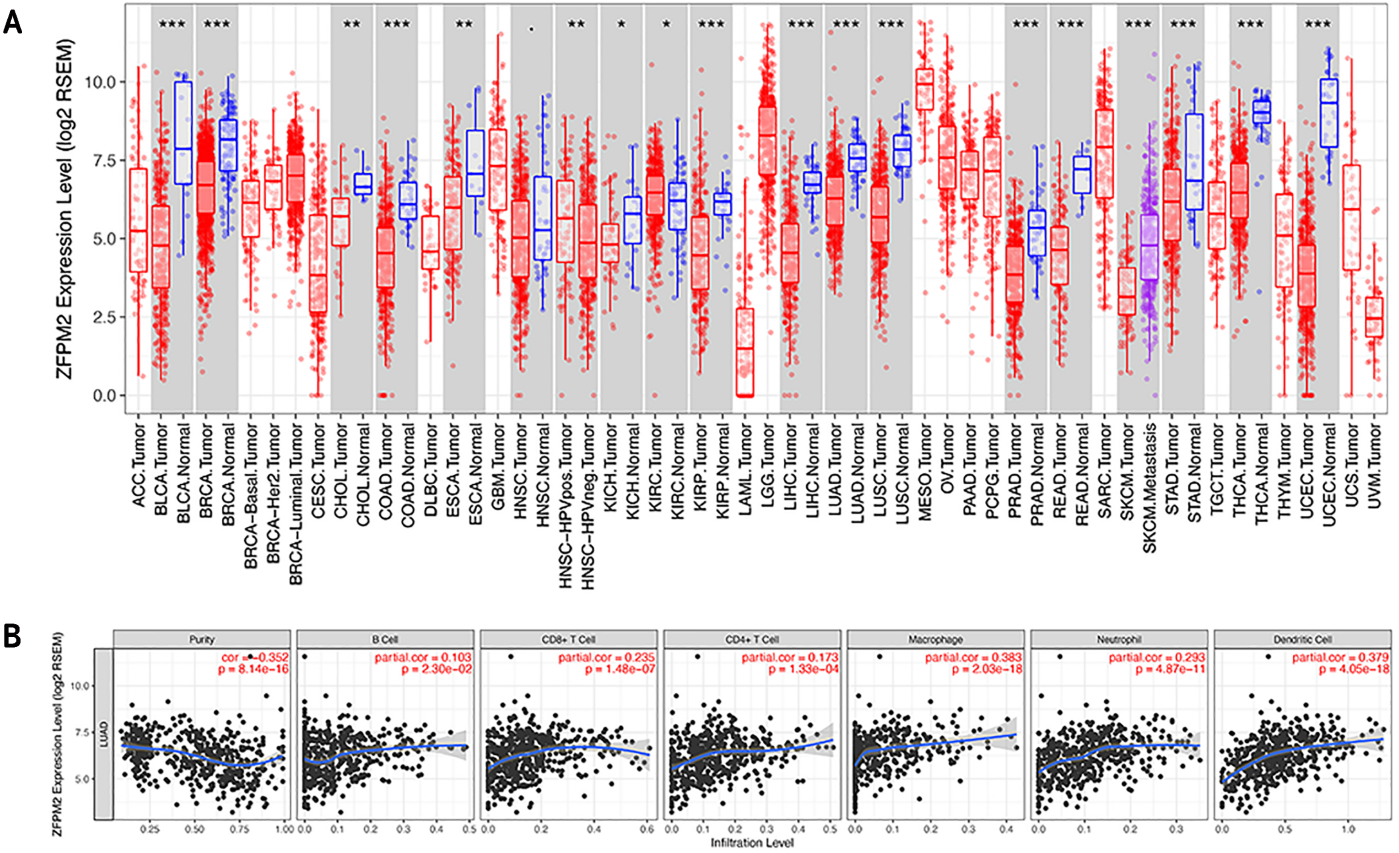
Correlations between ZFPM2 expression and immune infiltration level. (A) ZFPM2 expression levels of various human tumor types were validated using TIMER (*p<0.05, **p<0.01, ***p<0.001). The color red signified tumor tissues, blue signified normal tissues, and purple signified metastasis tissue. (B) The correlations between ZFPM2 expression and tumor purity, B cells, CD8+ T cells, CD4+ T cells, macrophages, neutrophils, or dendritic cells were analyzed.

To determine whether ZFPM2-AS1 was associated with tumor immune infiltration level, we mined data from the TIMER database. Since ZFPM2 is a potential target for ZFPM2-AS1, we found that ZFPM2 expression was significantly lower in most human tumors compared to the adjacent normal tissues, including LUAD and LUSC (Fig. 6A). ZFPM2-AS1 expression was significantly higher in NSCLC tissues compared to normal lung tissues (Fig. 1A), suggesting a possible negative correlation between ZFPM2-AS1 and ZFPM2. We also found a negative correlation between ZFPM2 expression and the immune infiltrate levels of tumor purity (*R* =  − 0.352, *P* = 8.14e^−16^) in LUAD (Fig. 6B). These results implied that ZFPM2 expression was significantly lower in LUAD and LUSC, as well as negatively correlated with tumor immune infiltration levels. We wanted to further verify the positive correlation between ZFPM2-AS1 and the immune infiltrating marker PD-L1 using Western blot.

### ZFPM2-AS1′s negative regulation of ZFPM2 expression and positive regulation of PD-L1 expression via the JAK-STAT and AKT pathways

**Figure 7:**
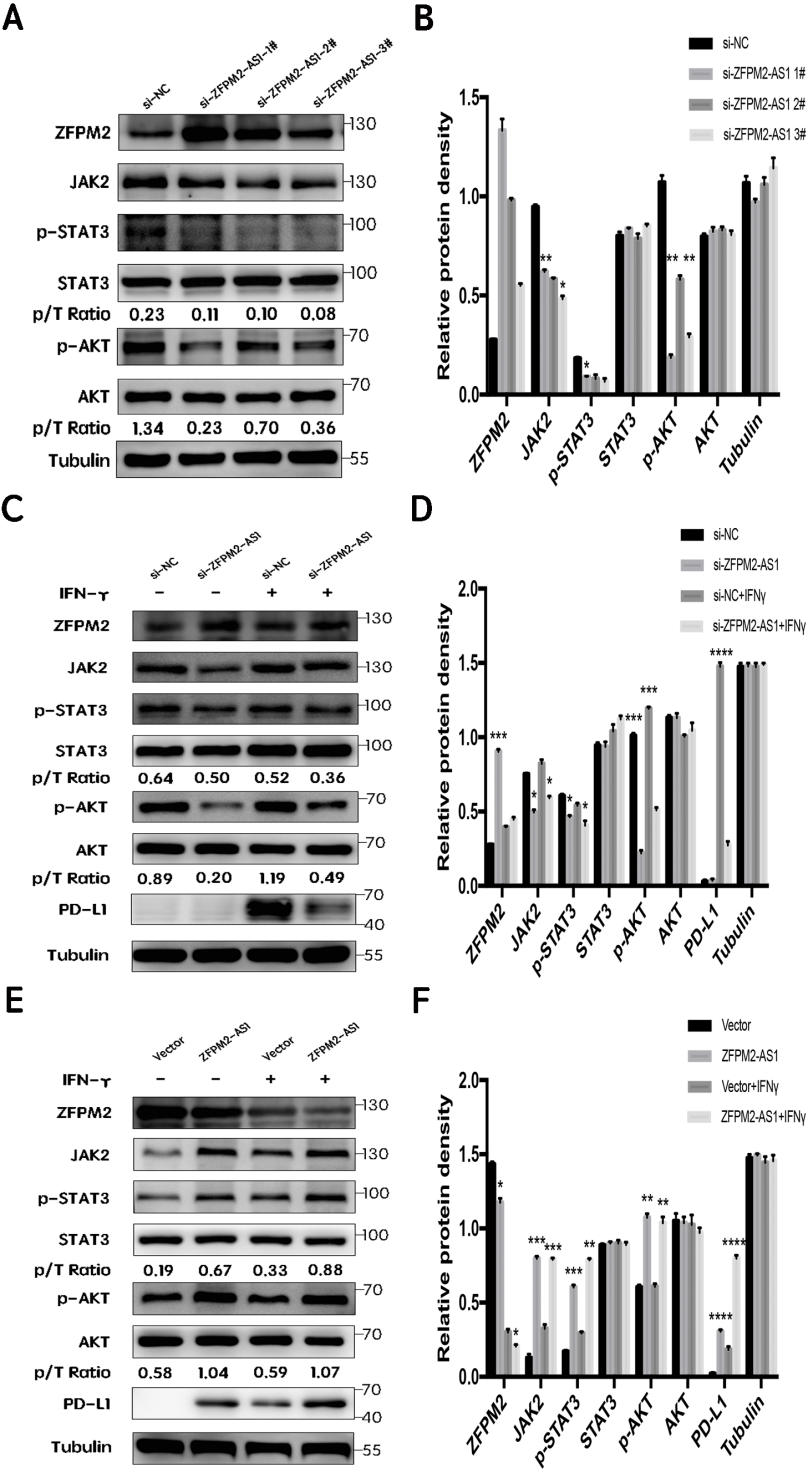
ZFPM2-AS1 positively regulated PD-L1 expression via the JAK-STAT and AKT pathways. (A, B) ZFPM2-AS1 was downregulated by three individual siRNAs. The relevant proteins were identified by Western blot during ZFPM2-AS1 knockdown (C, D) or overexpression, (E, F) with or without IFN-*γ* treatment.

First, we silenced ZFPM2-AS1 expression using three individual siRNAs in the A549 cells. The results showed a significant increase in ZFPM2 expression, indicating that ZFPM2-AS1 may negatively regulate ZFPM2 expression. Meanwhile, JAK2, p-STAT3, and p-AKT expression decreased in comparison to the control group. However, there was no significant differential STAT3 and AKT expression (Figs. 7A, 7B). We used siRNA to induce more significant ZFPM2 differences in the following experiment. We performed ZFPM2-AS1 silencing, both with and without IFN-*γ* exposure, to identify the differences between the JAK-STAT and AKT pathways. We found that in both the presence and absence of IFN-*γ*, ZFPM2-AS1 knockdown significantly upregulated ZFPM2 expression and downregulated JAK2, p-STAT3, and p-AKT expression. PD-L1 expression was distinctly inhibited when ZFPM2-AS1 was silenced by IFN- *γ* stimulation (Figs. 7C, 7D). Furthermore, ZFPM2-AS1 overexpression downregulated ZFPM2 expression and upregulated JAK2, p-STAT3, p-AKT, and PD-L1 expression in the presence or absence of IFN-*γ* (Figs. 7E, 7F). Ultimately, we determined that ZFPM2-AS1 negatively regulated ZFPM2 expression and positively regulated PD-L1 expression through the JAK-STAT and AKT pathways.

## Discussion

Recent studies have shown that lncRNAs can operate as biomarkers in the diagnosis, therapy, and prognosis of various malignant tumors (Heery et al., 2017; Chandra Gupta & Nandan Tripathi, 2017). Our results indicated that ZFPM2-AS1 was upregulated in NSCLC specimens and cell lines when compared to the control groups. Additionally, we found a correlation between ZFPM2-AS1 and poor survival in TCGA. When looking at clinical statistics, we found that higher ZFPM2-AS1 expression levels were positively correlated with larger tumor sizes and later TNM stages. These findings suggest that ZFPM2-AS1 may be a potential novel biomarker for NSCLC. Our study also verified that the downregulation of ZFPM2-AS1 expression significantly inhibited the proliferation, migration, and invasion of A549 and H460 cells, suggesting that ZFPM2-AS1 frequently played an oncogenic role. Similarly, Han et al. (2020) reported that ZFPM2-AS1 facilitated the proliferation, invasion, and epithelial-to-mesenchymal transition in LUAD, and that UPF1 de-stabilized the ZFPM2 mRNA level negatively regulated by ZFPM2-AS1. Their observation of a negative correlation between ZFPM2-AS1 and ZFPM2 was also consistent with our findings.

ZFPM2-AS1 has been reported to induce p53 destabilization stabilizing MIF, leading to the progression of gastric cancer (Kong et al., 2018). However, the connection between ZFPM2-AS1 and the JAK/STAT signal pathway has not been explored. In our study, we found that ZFPM2-AS1 positively regulated the expression of JAK2, p-STAT3, and PD-L1 in A549 cells. JAK kinase phosphorylated STAT C-terminus Tyr705 in STAT3, initiated by the binding of IL-6 to its specific receptor and the activation of phosphorylated JAK. A different study reported that the activation of p-STAT3 enhanced cell proliferation, metastasis, and angiogenesis in multiple cancers including NSCLC (Dutta et al., 2014). PI3K/AKT, RAS/MAPK, and JAK/STAT3 are three major downstream activated EGFR phosphorylation pathways (Mitsudomi & Yatabe, 2007). Our results revealed that ZFPM2-AS1 also positively regulated p-AKT expression, confirming the existence of crosstalk between the JAK2-STAT3 and PI3K-AKT pathways.

Previous research has shown that PD-L1 expression is involved in two main mechanisms: the innate immune escape, which is associated with multiple oncogenes, and the adaptive immune escape, which consists of various tumor microenvironment inflammatory factors (Zhang et al., 2013; Pardoll, 2012; Rech & Vonderheide, 2013; Taube et al., 2012). Wang et al. (2019a) and Wang et al. (2019b) reported that the lncRNA MALAT1 regulated PD-L1 by sponging miR-195 in diffuse large B cell lymphoma, affecting PD-L1’s proliferation, apoptosis, migration, and immune escape capacities. Notably, our study confirmed that ZFPM2-AS1 knockdown decreased PD-L1 expression in the presence of IFN-*γ*, suggesting that ZFPM2-AS1 may be a potential target during PD-L1 immunotherapy. However, our study’s exploration of how ZFPM2-AS1 regulates PD-L1 in NSCLC cells was limited, and this mechanism should be thoroughly studied in future investigations.

ZFPM2’s role as a cytokine has been shown to play a crucial role in the regulation of the immune system (Nath et al., 2019). Our study demonstrated that ZFPM2 expression was negatively regulated by ZFPM2-AS1, indicating that ZFPM2 may also be correlated with tumor immune infiltration. The TIMER database showed that ZFPM2 expression had a negative correlation with the immune infiltrating levels of tumor purity in LUAD, which was consistent with our initial hypothesis.

However, we found that ZFPM2-AS1 was downregulated in H1299 and H292 cell lines, revealing that low ZFPM2-AS1 expression may progress cancer using other signal pathways. Further investigations are needed to determine how ZFPM2-AS1 regulates NSCLC function in these cell lines.

## Conclusion

In this study, we found that the lncRNA ZFPM2-AS1 functioned as an oncogene by promoting the proliferation, migration, and invasion of NSCLC cells. Furthermore, we determined that ZFPM2-AS1 positively regulated PD-L1 expression via the JAK-STAT and AKT pathways in A549 cell lines.

##  Supplemental Information

10.7717/peerj.10225/supp-1 Supplemental Information 1 Specific primers for qRT-PCR and siRNA Smart Silencer sequences

10.7717/peerj.10225/supp-2 Supplemental Information 2 We checked if the JAK inhibitor can abolish the proliferation of ZFPM2-AS1 in Fig. 3C

10.7717/peerj.10225/supp-3 Supplemental Information 3 The effect of p-JAK2 primary antibody in Western Blot was not satisfied

10.7717/peerj.10225/supp-4 Supplemental Information 4 Raw data for Fig. 1A

10.7717/peerj.10225/supp-5 Supplemental Information 5 Raw data for Fig. 2

10.7717/peerj.10225/supp-6 Supplemental Information 6 Raw data for Fig. 3

10.7717/peerj.10225/supp-7 Supplemental Information 7 Raw data for Fig. 4

10.7717/peerj.10225/supp-8 Supplemental Information 8 Raw data for Fig. 5

10.7717/peerj.10225/supp-9 Supplemental Information 9 Raw data for Fig. 6A and Fig. 6B

10.7717/peerj.10225/supp-10 Supplemental Information 10 Raw data for Fig. 7

## Additional Information and Declarations

### Data Availability

The following information was supplied regarding data availability: The raw measurements are available in the Supplemental Files.
