# Supplementary material for: ZFPM2-AS1 promotes the proliferation, migration, and invasion of human non-small cell lung cancer cells involving the JAK-STAT and AKT pathways
Source: PeerJ. 2020 Oct 26;8:e10225. doi: 10.7717/peerj.10225 (PMC7594634; doi:10.7717/peerj.10225)
Supplement: Supplemental Information 1 [file peerj-08-10225-s001.docx]

1.Specific primers for qRT-PCR

| Primer | sequence(5' to 3') |
| --- | --- |
| GAPDH Forward | CAGGAGGCATTGCTGATGAT |
| GAPDH Reverse | GAAGGCTGGGGCTCATTT |
| HS-RPS18 Forward | ATCCTCAGTGAGTTCTCCCG |
| HS-RPS18 Reverse | CTTTGCCATCACTGCCATTA |
| ZFPM2-AS1 Forward | GGTGGCACCTGAAATCACAGA |
| ZFPM2-AS1 Reverse | TGCAAGATGACGCTCAGTCG |
| U1 Forward | CCATGATCACGAAGGTGGTTT |
| U1 Reverse | ATGCAGTCGAGTTTCCCACAT |

2.Sequences for siRNA Smart Silencer

| siRNA Smart Silencer | Target sequence |
| --- | --- |
| siRNA-1 | GCAGAGTTGCACAGAAGAA |
| siRNA-2 | GAAGGAAGTGGATAAACAA |
| siRNA-3 | CAAAGAATTTGAACCTCAA |
| Antisense oligonucleotides-1 | TTGGGCAATTAATTAAGCCA |
| Antisense oligonucleotides-2 | GTAGTACGCATTCTACCTTT |
| Antisense oligonucleotides-3 | GATTCTCACTGTGTTACCTA |
