# Supplementary figures and images for: ZFPM2-AS1 promotes the proliferation, migration, and invasion of human non-small cell lung cancer cells involving the JAK-STAT and AKT pathways
Source: PeerJ. 2020 Oct 26;8:e10225. doi: 10.7717/peerj.10225 (PMC7594634; doi:10.7717/peerj.10225)

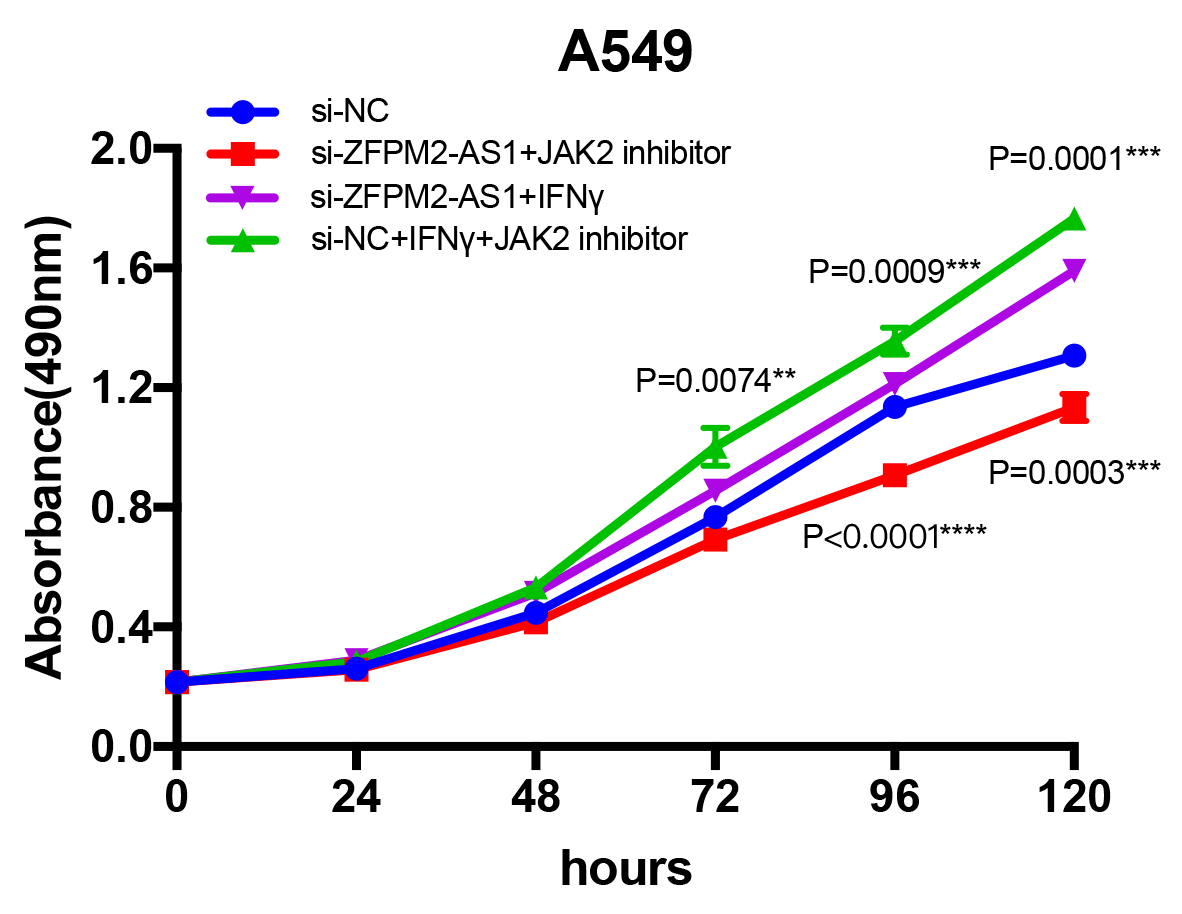

Supplement: Supplemental Information 2 [file peerj-08-10225-s002.png]

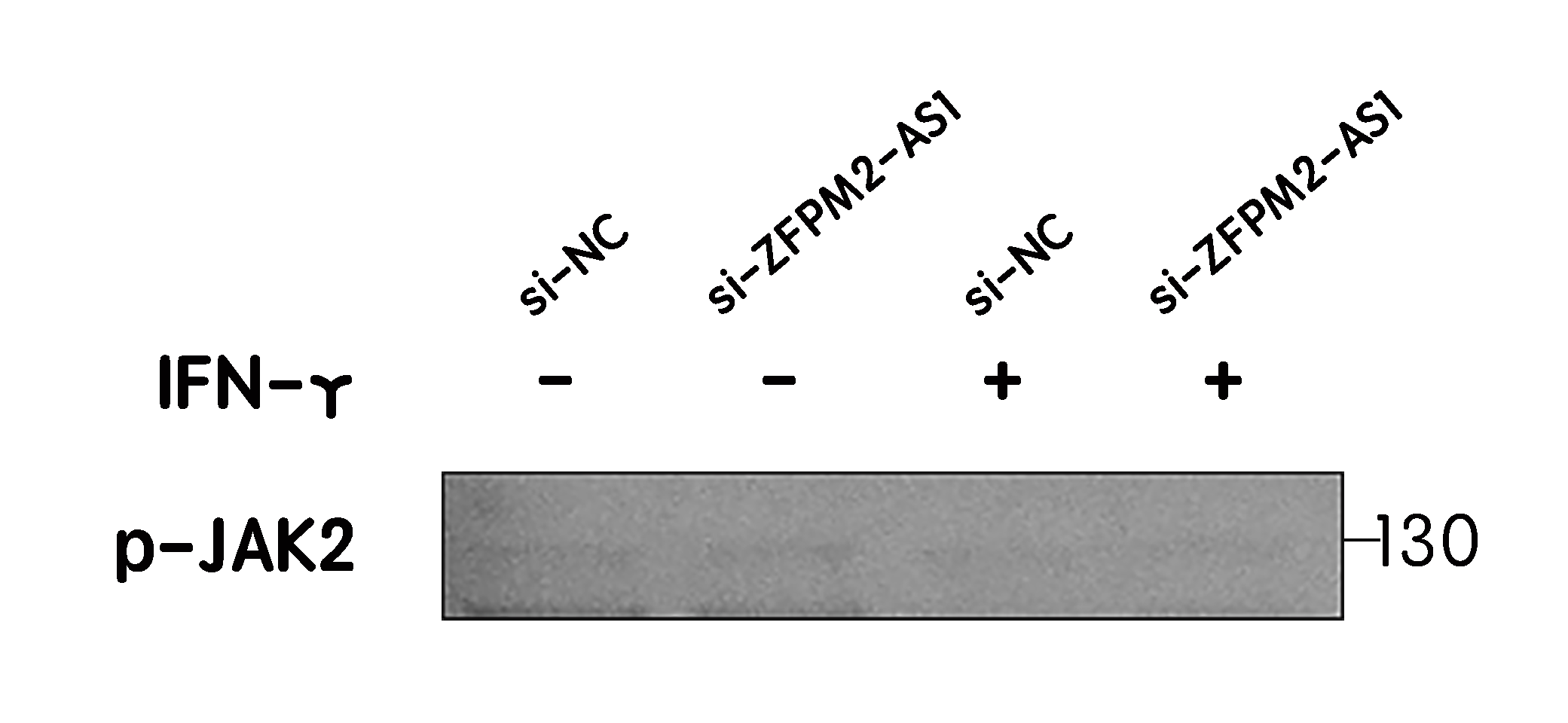

Supplement: Supplemental Information 3 [file peerj-08-10225-s003.png]

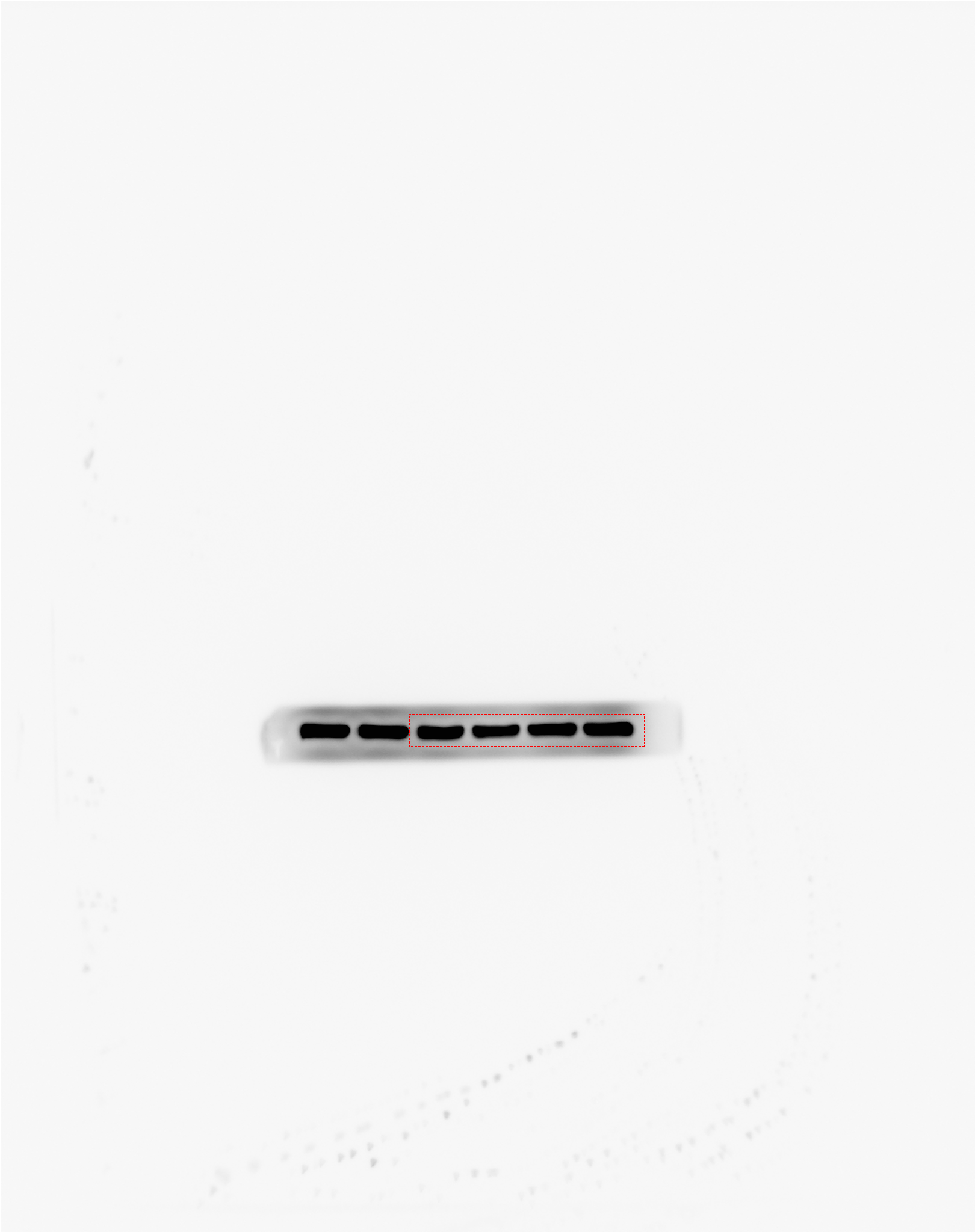

Supplement: Supplemental Information 9 [file peerj-08-10225-s009.zip › fig6A/figure6A-tubulin]

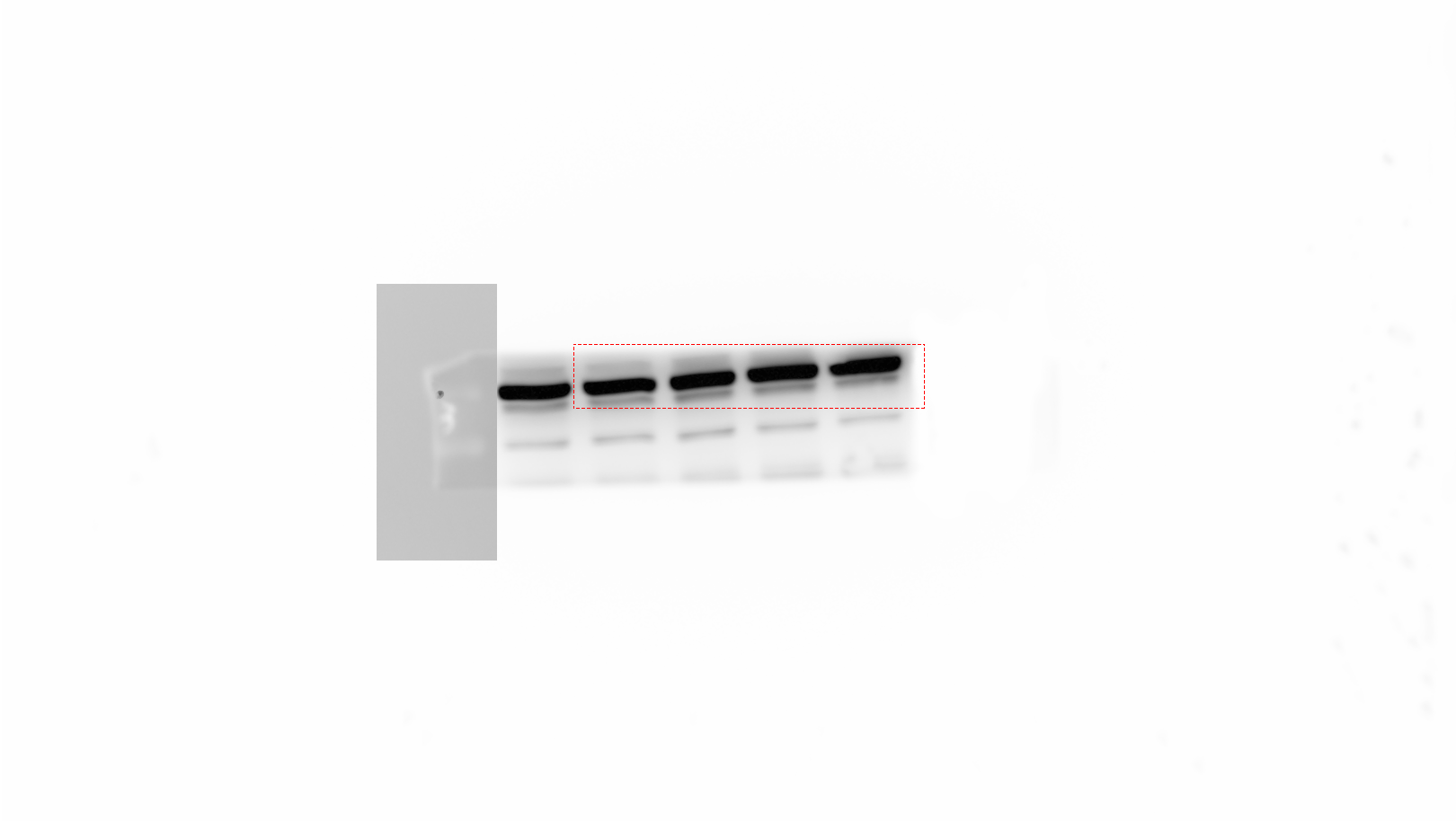

Supplement: Supplemental Information 9 [file peerj-08-10225-s009.zip › fig6A/figure6A-STAT3.bmp]

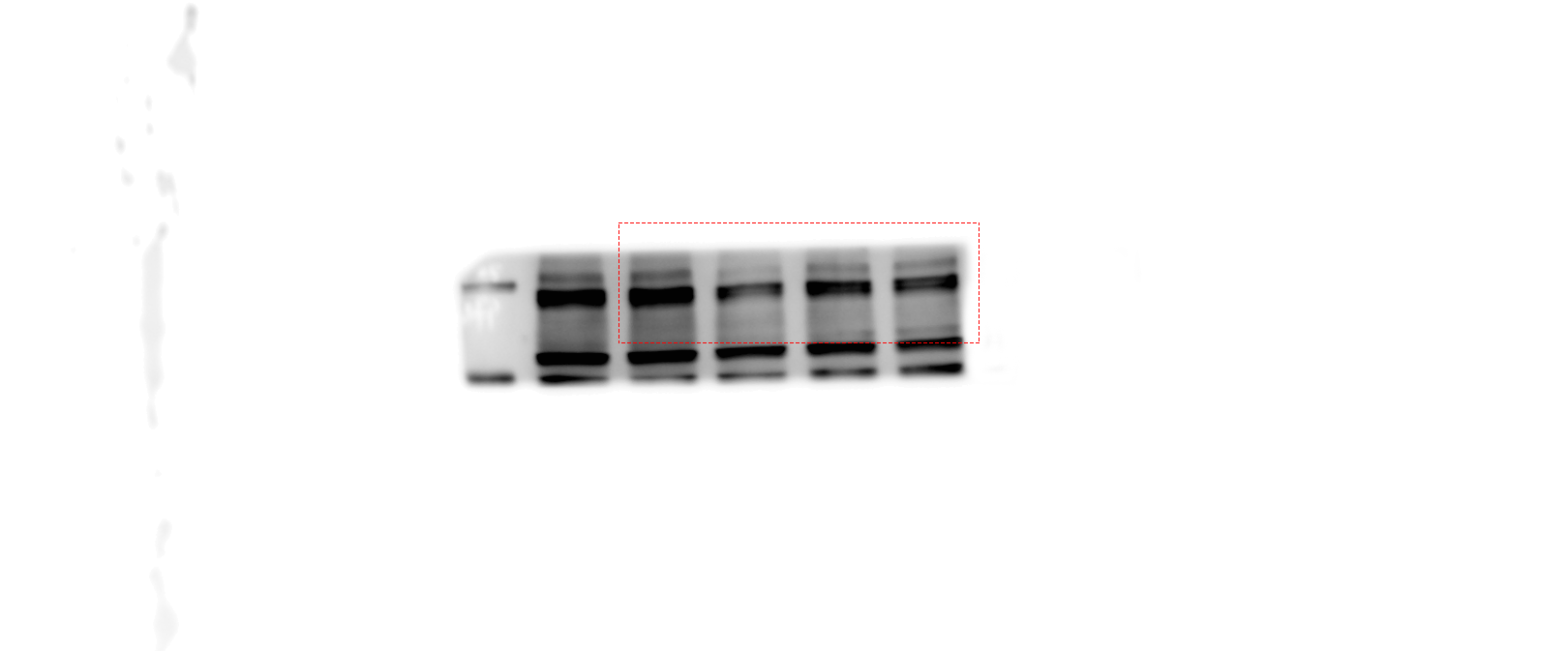

Supplement: Supplemental Information 9 [file peerj-08-10225-s009.zip › fig6A/figure6A-pAKT.bmp]

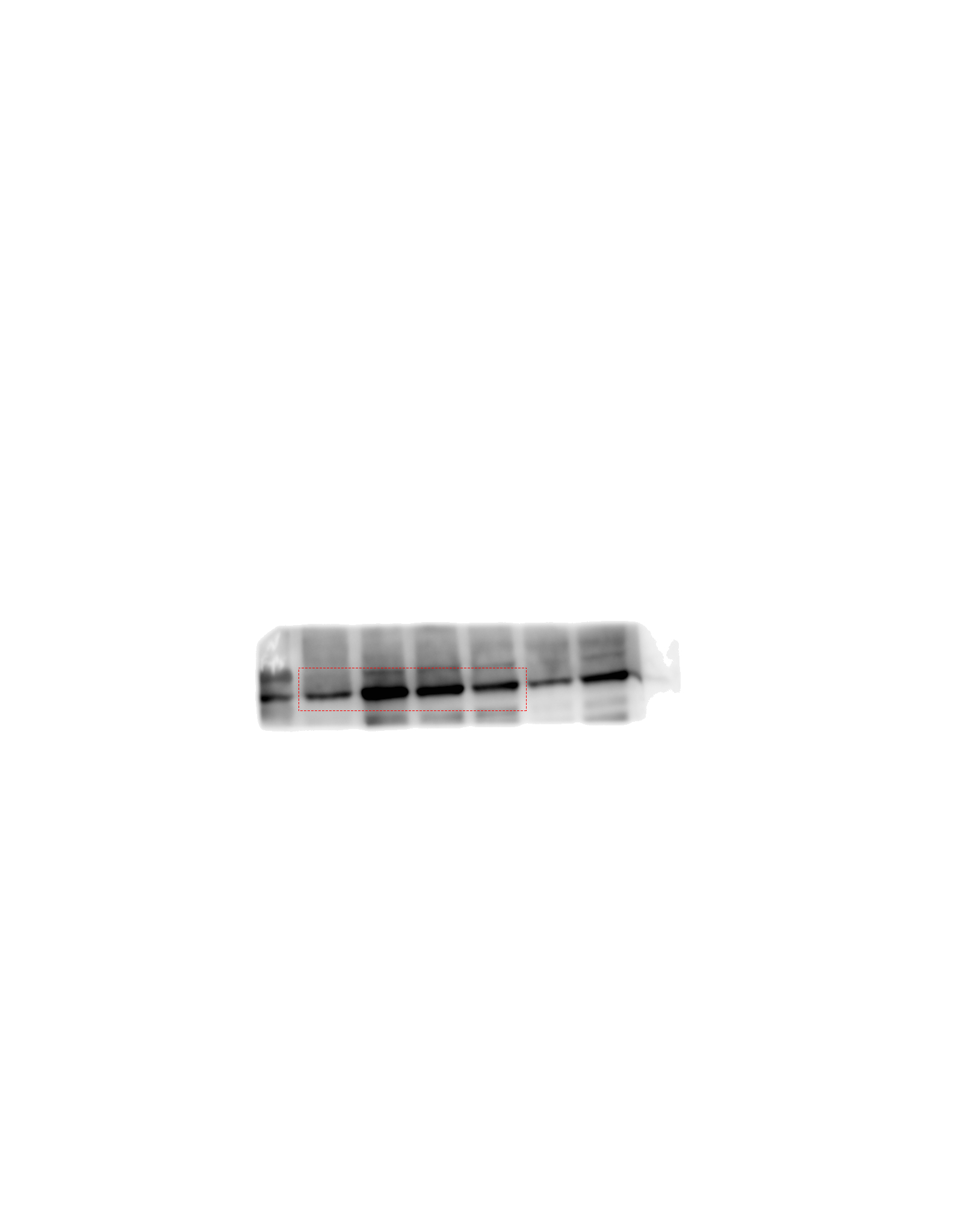

Supplement: Supplemental Information 9 [file peerj-08-10225-s009.zip › fig6A/figure6A-ZFPM2.bmp]

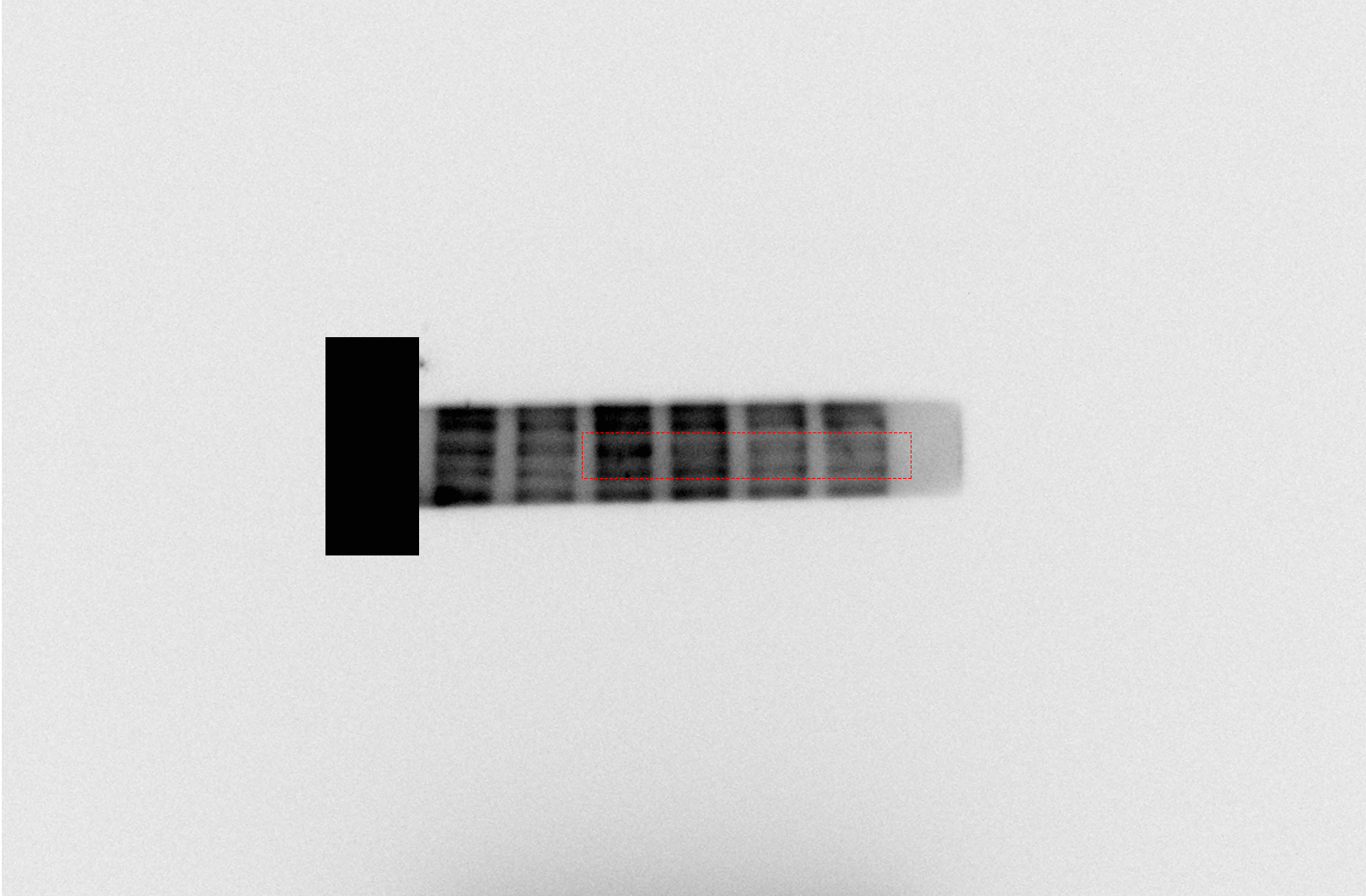

Supplement: Supplemental Information 9 [file peerj-08-10225-s009.zip › fig6A/figure6A-pSTAT3.bmp]

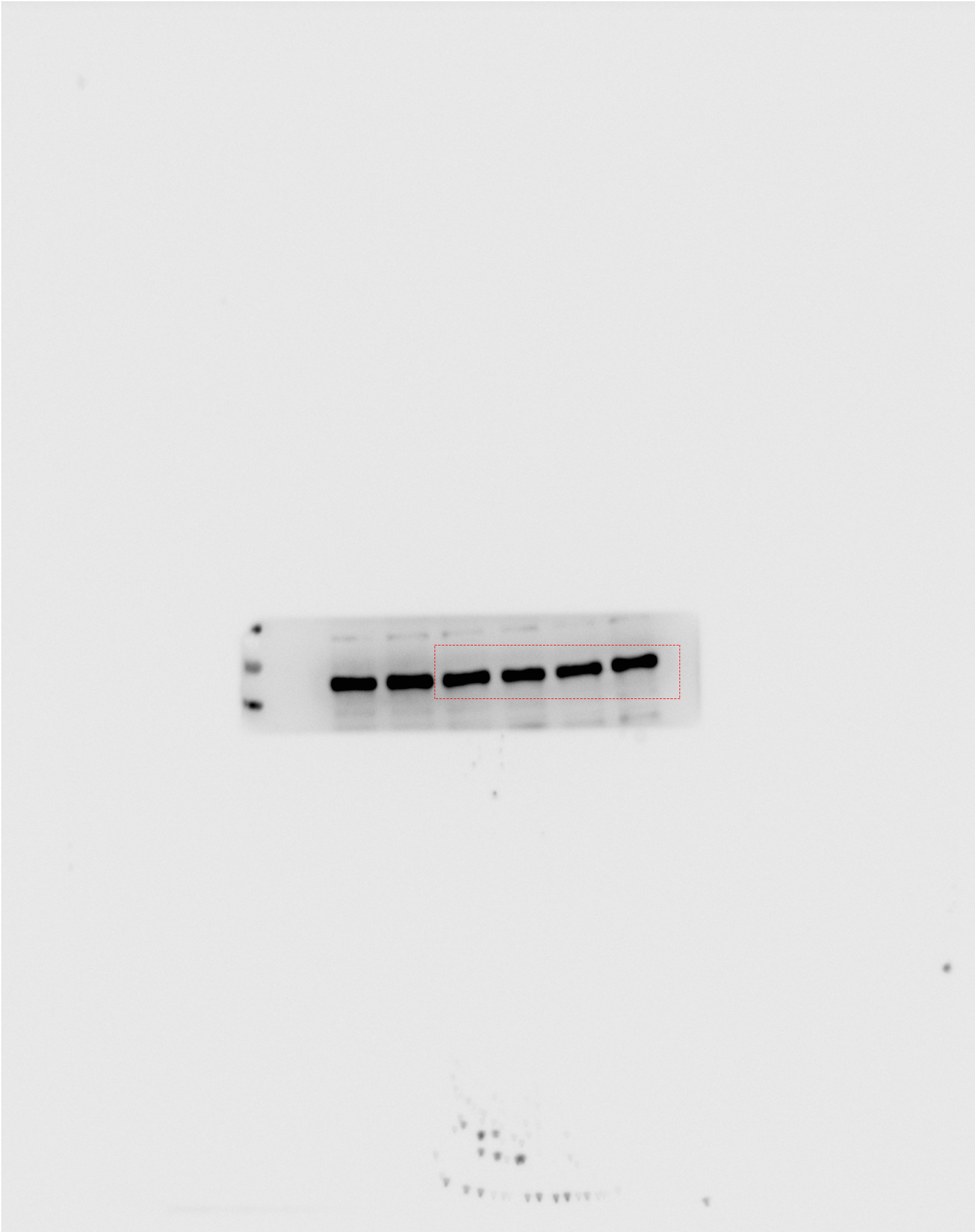

Supplement: Supplemental Information 9 [file peerj-08-10225-s009.zip › fig6A/figure6A-AKT.bmp]

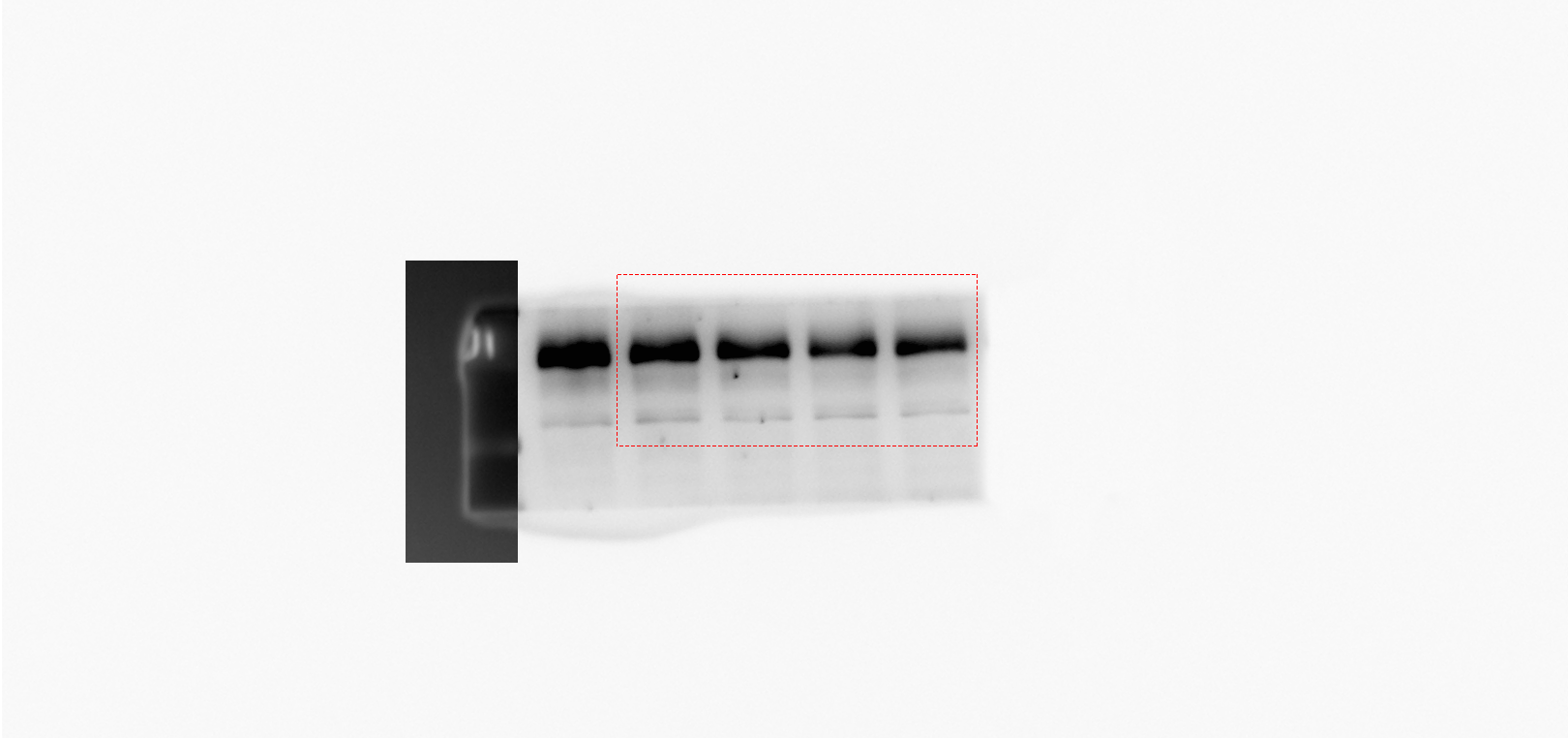

Supplement: Supplemental Information 9 [file peerj-08-10225-s009.zip › fig6A/figure6A-JAK2.tif]

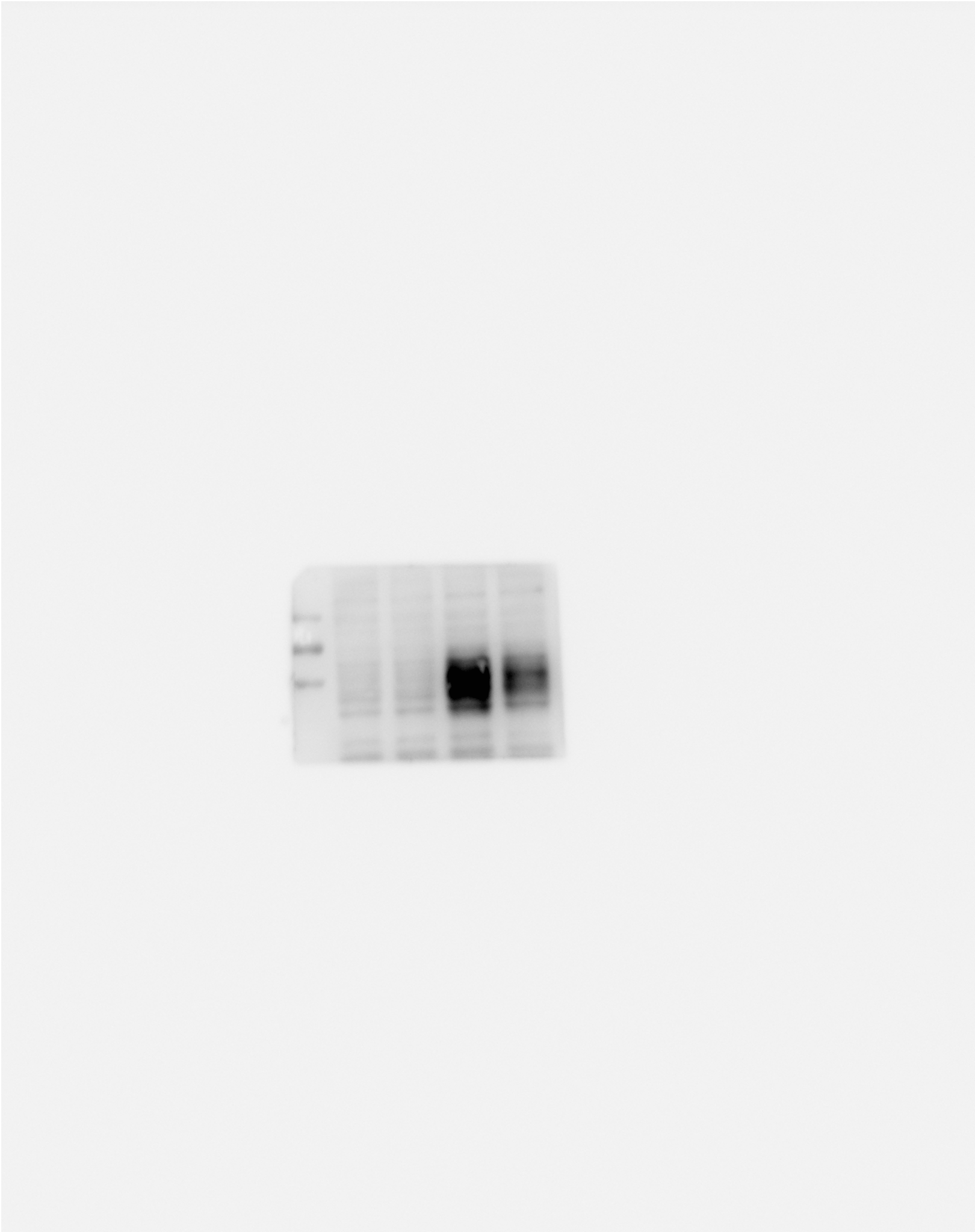

Supplement: Supplemental Information 9 [file peerj-08-10225-s009.zip › fig6B/figure6B--PDL1.bmp]

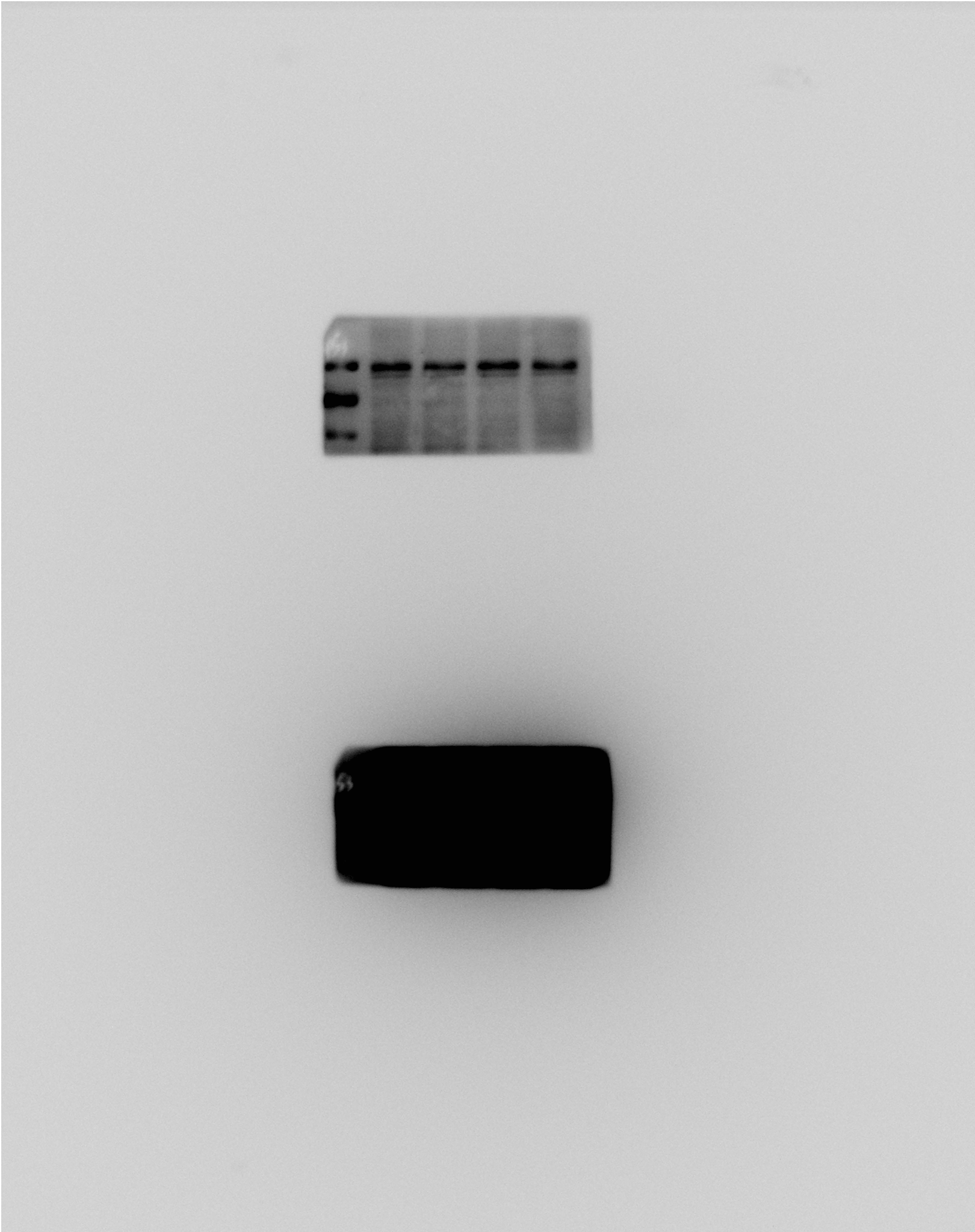

Supplement: Supplemental Information 9 [file peerj-08-10225-s009.zip › fig6B/figure6B-pSTAT3.bmp]

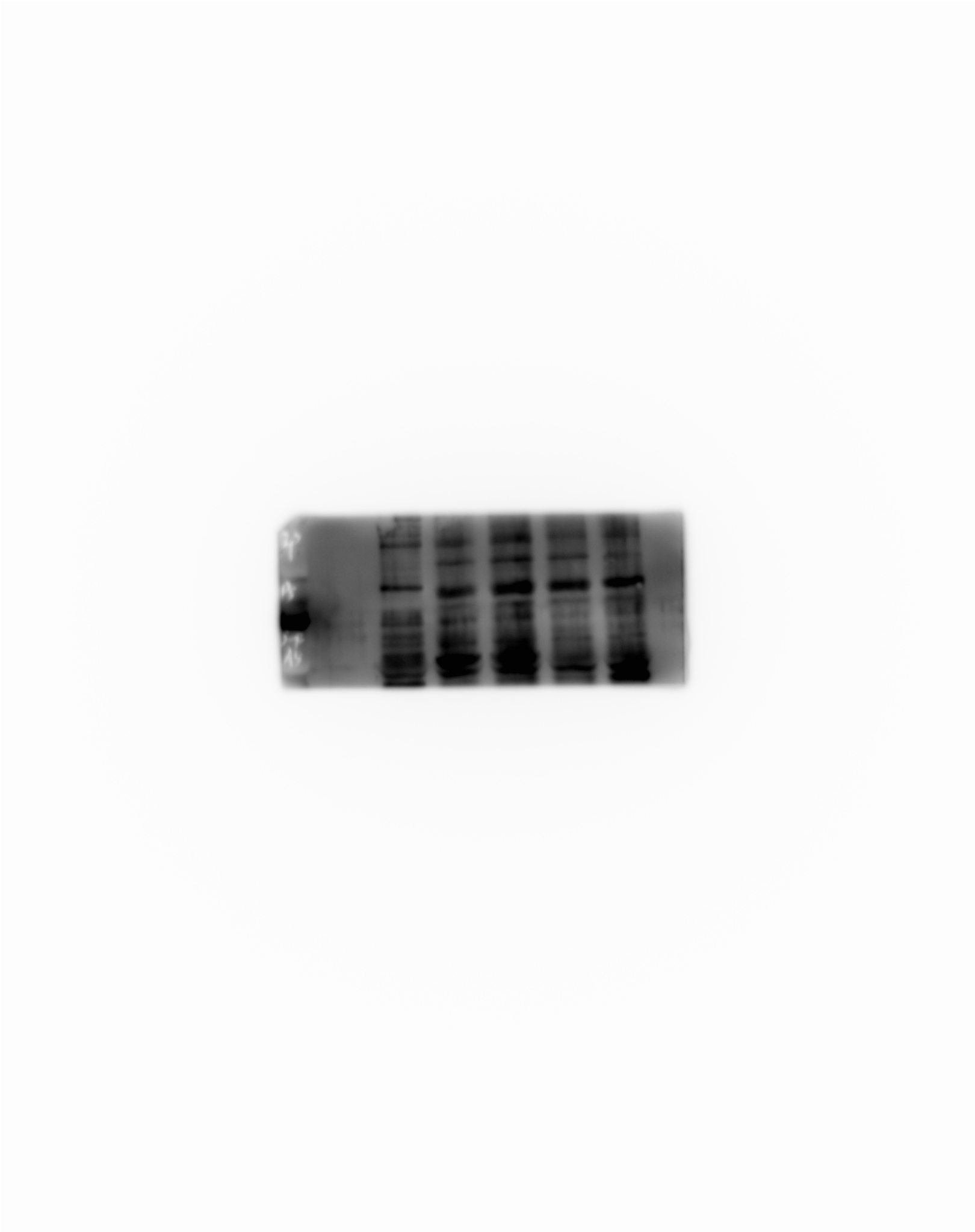

Supplement: Supplemental Information 9 [file peerj-08-10225-s009.zip › fig6B/figure6B-ZFPM2.bmp]

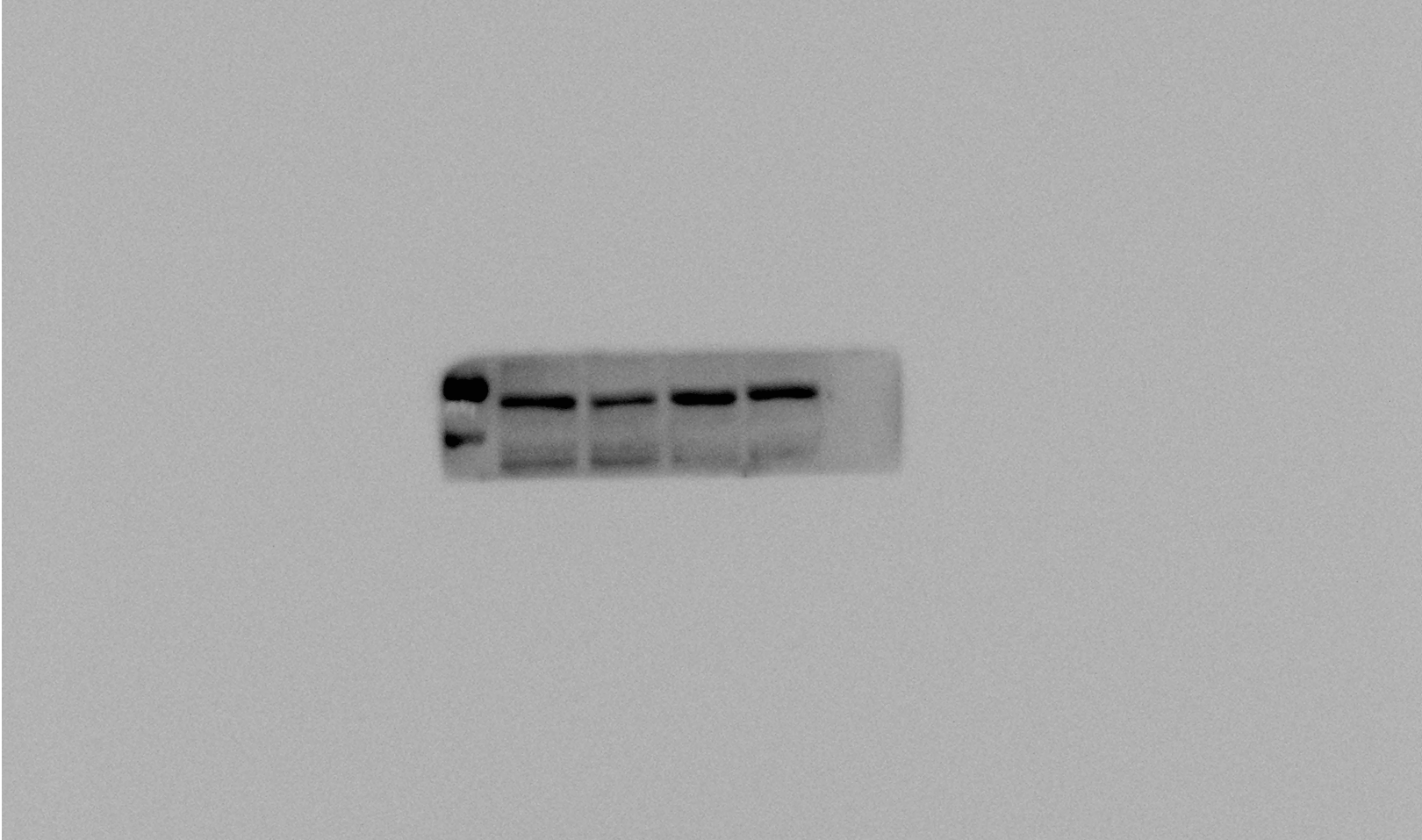

Supplement: Supplemental Information 9 [file peerj-08-10225-s009.zip › fig6B/figure6B-JAK2.bmp]

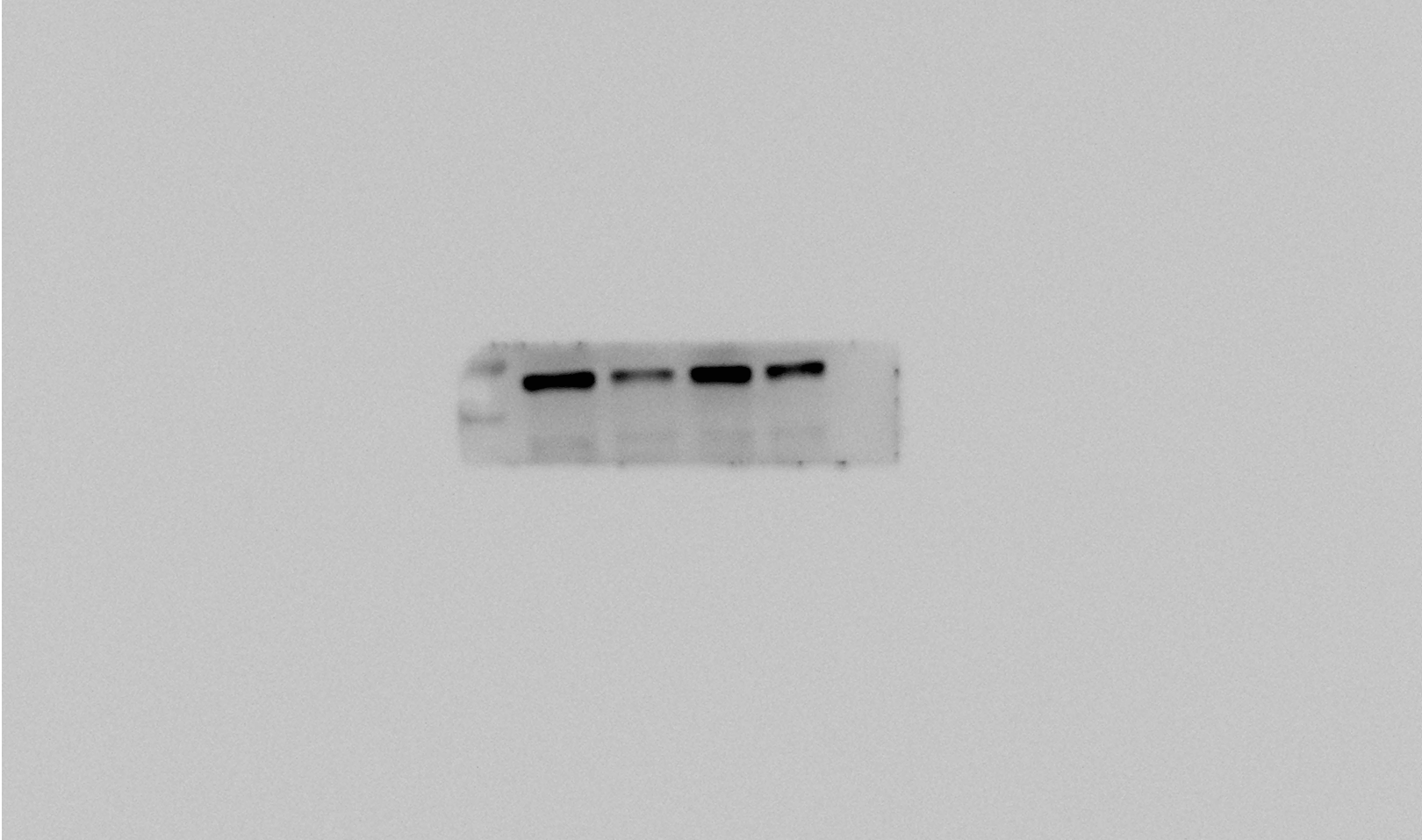

Supplement: Supplemental Information 9 [file peerj-08-10225-s009.zip › fig6B/figure6B-pAKT.bmp]

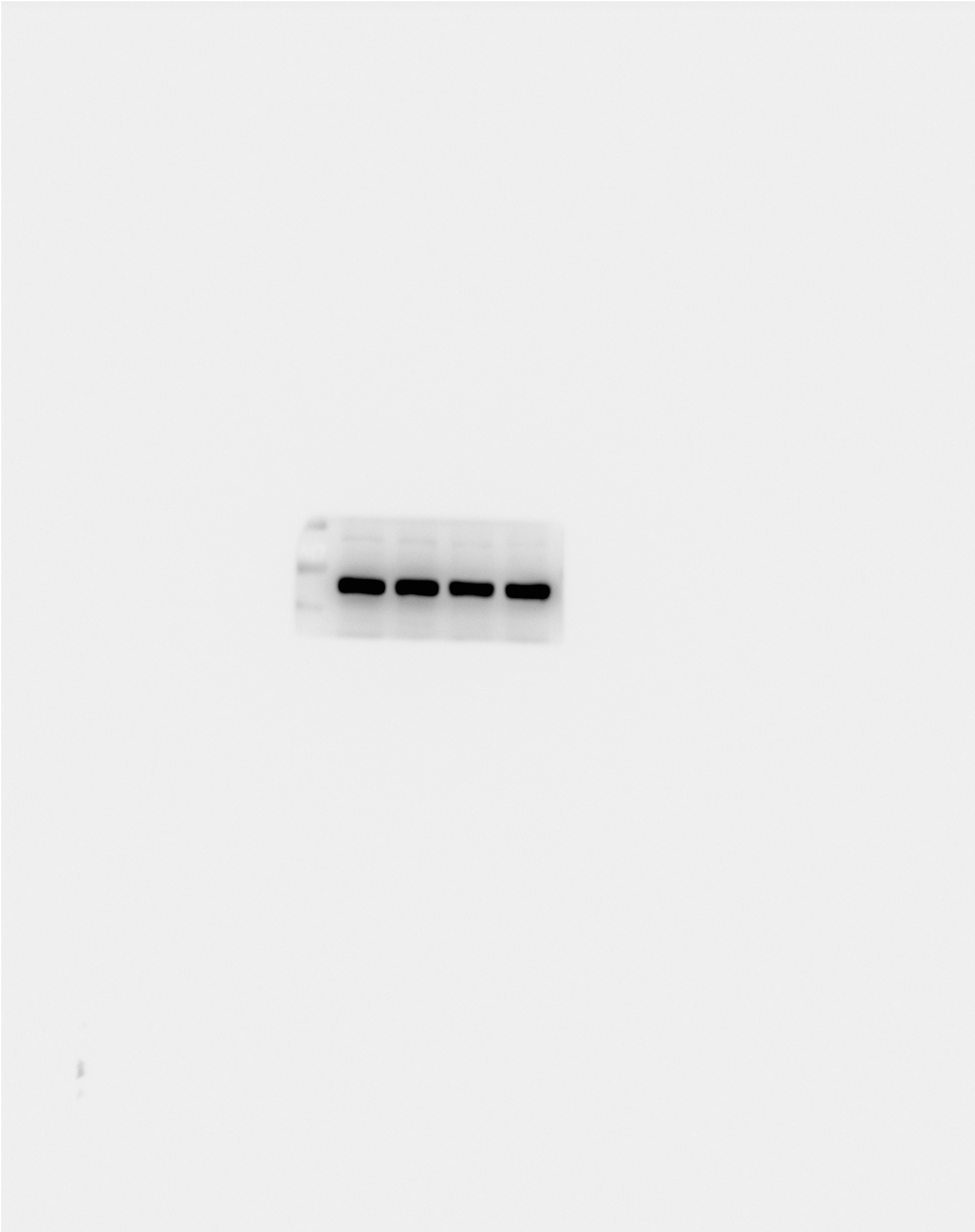

Supplement: Supplemental Information 9 [file peerj-08-10225-s009.zip › fig6B/figure6B-AKT.bmp]

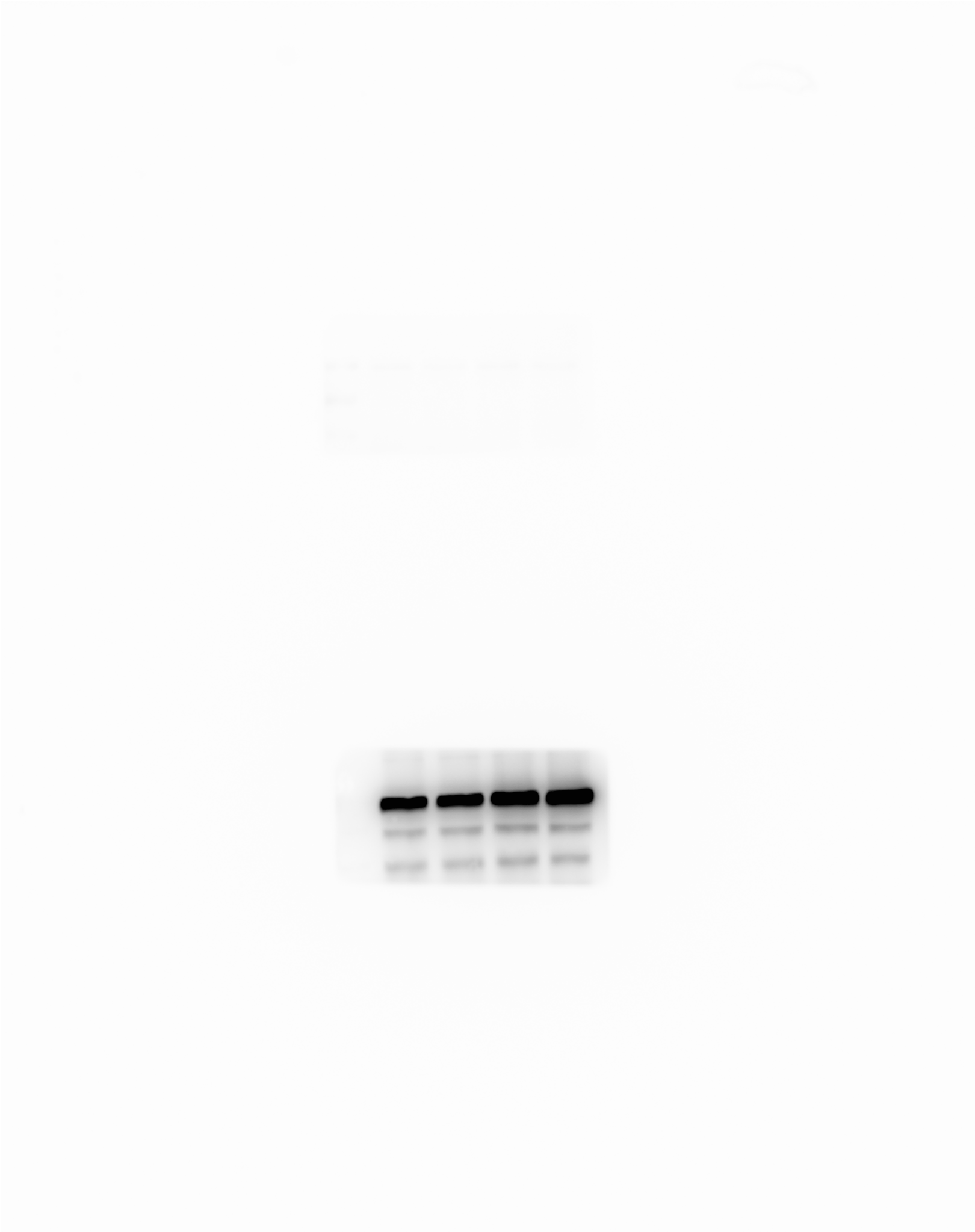

Supplement: Supplemental Information 9 [file peerj-08-10225-s009.zip › fig6B/figure6B-STAT3.bmp]

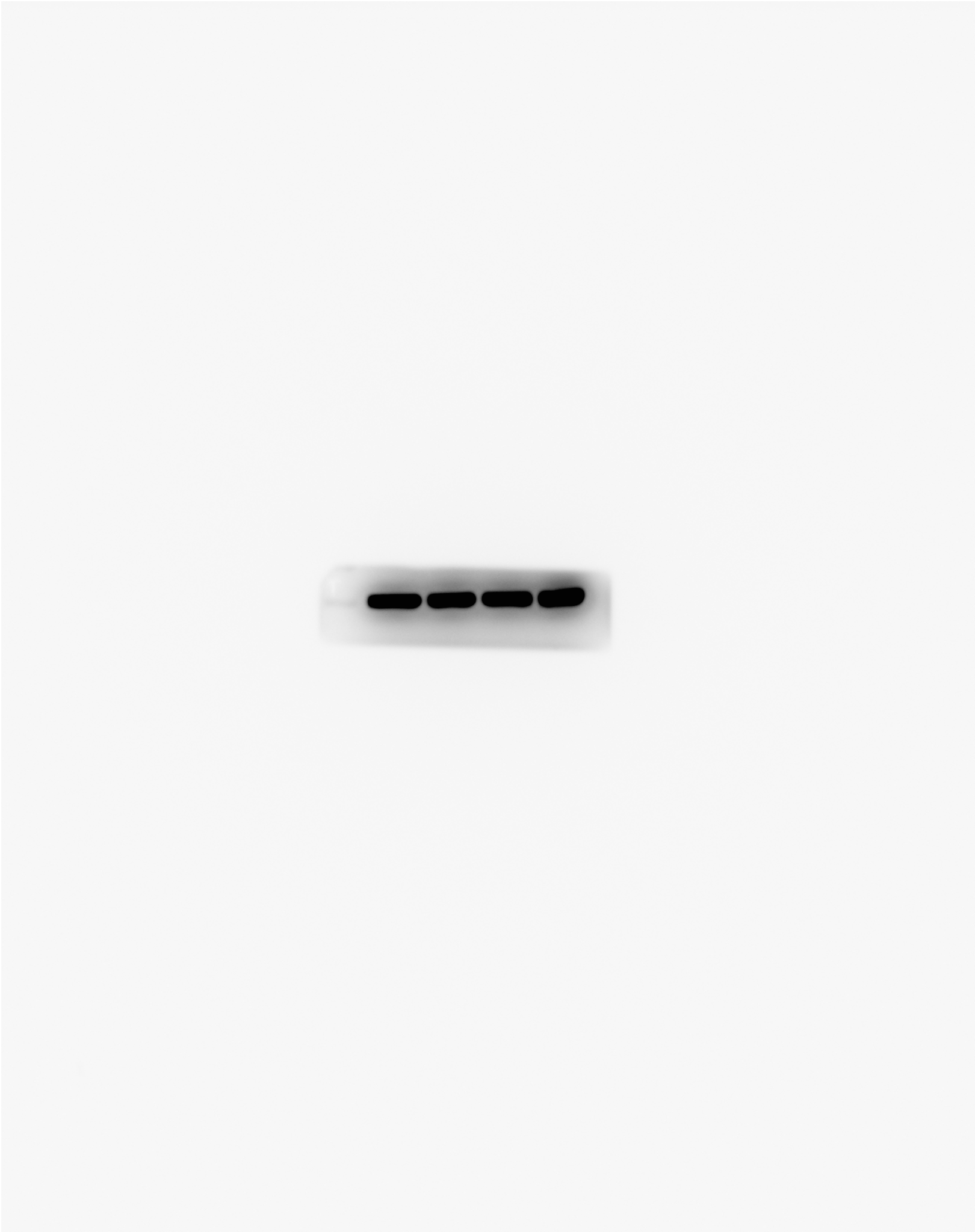

Supplement: Supplemental Information 9 [file peerj-08-10225-s009.zip › fig6B/figure6B--tubulin.bmp]
